# Comparison of Gut Microbiota Diversity and Predicted Functions Between Healthy and Diseased Captive *Rana dybowskii*

**DOI:** 10.3389/fmicb.2020.02096

**Published:** 2020-09-01

**Authors:** Qing Tong, Li-Yong Cui, Xiao-Peng Du, Zong-Fu Hu, Jia Bie, Jian-Hua Xiao, Hong-Bin Wang, Jian-Tao Zhang

**Affiliations:** ^1^College of Veterinary Medicine, Northeast Agricultural University, Harbin, China; ^2^Jiamusi Branch of Heilongjiang Academy of Forestry Sciences, Jiamusi, China

**Keywords:** bacterial community, dysbiosis, diarrhoeic, functional prediction, microbiome

## Abstract

The gut microbiota plays a key role in host health, and disruptions to gut bacterial homeostasis can cause disease. However, the effect of disease on gut microbiota assembly remains unclear and gut microbiota-based predictions of health status is a promising yet poorly established field. Using Illumina high-throughput sequencing technology, we compared the gut microbiota between healthy (HA and HB) and diarrhoeic (DS) *Rana dybowskii* groups and analyzed the functional profiles through a phylogenetic investigation of communities by reconstruction of unobserved states (PICRUSt) analysis. In addition, we estimated the correlation between gut microbiota structures and predicted the functional compositions. The results showed significant differences in the phylogenetic diversity (Pd), Shannon, and observed richness (Sobs) indices between the DS and HB groups, with significant differences observed in the gut microbiota composition between the DS group and the HA and HB groups. Linear discriminant analysis (LDA) effect size (LEfSe) results revealed that Proteobacteria were significantly enriched in the DS group; Bacteroidetes were significantly enriched in the HA and HB groups; and *Aeromonas*, *Citrobacter*, *Enterococcus*, *Hafnia-Obesumbacterium*, *Morganella*, *Lactococcus*, *Providencia*, *Vagococcus*, and *Staphylococcus* were significantly enriched in the DS group. Venn diagrams revealed that there were many more unique genera in the DS group than the HA and HB groups. Among 102 sensitive species selected using the indicator method, 33 indicated a healthy status and 69 (e.g., *Acinetobacter*, *Aeromonas*, *Legionella*, *Morganella*, *Proteus*, *Providencia*, *Staphylococcus*, and *Vagococcus*) indicated a diseased status. There was a significant and positive association between the composition and functional composition of the gut microbiota, thus indicating low functional redundancy of the frog gut bacterial community. *Rana dybowskii* disease was associated with changes in the gut microbiota, which subsequently disrupted bacterial-mediated functions. The results of this study can aid in revealing the effect of the *R. dybowskii* gut microbiota on host health and provide a basis for elucidating the mechanism of the occurrence of *R. dybowskii* disease.

## Introduction

The gut microbiota represents a significant microbiota in the host (Rudi et al., 2018), and in recent years, numerous studies have investigated how various factors may impact the gut bacterial community, such as antibiotic use, health, diet, nutrition, and age (Pascoe et al., 2017). Most studies on the gut bacterial community have been conducted in mammals, especially humans and laboratory rodents (Kohl and Yahn, 2016). In contrast, there is a paucity of information regarding the gut bacterial community of amphibians (Kohl and Yahn, 2016; Jiménez and Sommer, 2017).

Currently, the interplay between the gut microbiota and host health is an important topic that has received a great deal of interest (Xiong et al., 2017). Studies have shown that different structures and compositions of the gut microbiota can affect the nutritional metabolism and sensitivity to external pathogen infection of the host (Colombo et al., 2015). Infection with pathogenic microorganisms can alter the composition of the gut microbiota, which in turn destroys the normal function of the gut microorganisms and leads to disease (Qi et al., 2019). The microbiome has a profound effect on host health and disease (Ma et al., 2019). However, previous studies on amphibians have not focused on the gut microbiota at the community level but rather focused on one or a few potential single pathogens from diseased individuals (Gomez et al., 2017; Jiménez and Sommer, 2017). In recent years, numerous studies have shown that the occurrence of disease is caused by the synergy of a variety of pathogens (Mosser et al., 2015). However, few studies have used changes in the gut microbiota to assess the health of amphibians.

Functional predictions can link the structure of the gut microbiome with the function of the gut microbiota and thus may better clarify the pathogenesis (Xiong et al., 2017; Yu et al., 2018). Several studies have shown that captivity may increase the relative abundance of some potential pathogenic bacteria in the amphibian gut microbiota (Xiang et al., 2018; Tong et al., 2019). Studies have been conducted on the correlation between disease occurrence and the composition of the gut microbiome in some aquaculture animals (e.g., shrimp) (Xiang et al., 2018; Tong et al., 2019). However, the current study focuses on the correlation between disease occurrence and the composition of the gut microbiota and on obtaining functional information on how community changes affect microbial correlations (Yu et al., 2018). Because microbiota has high functional redundancy, the extent to which changes in the gut microbiota affect different functions remains unclear (Yu et al., 2018). In recent years, due to the rapid development of bioinformatics, it has become possible to predict the functions corresponding to different bacteria (Yu et al., 2018). A previous study used PICRUSt to perform functional predictions regarding the core bacterial communities on the skin of *Plethodon cinereus* and showed that the core bacteria were closely linked to immunomodulation (Loudon et al., 2014). Yu et al. (2018) performed functional predictions of metabolic processes (such as antibacterial immunity, lysosomes, and peroxidase) and noted that they were weakened in diseased animals (Yu et al., 2018). Thus, functional prediction can link the structure of the gut microbiota with their function and clarify pathogenesis. However, few studies have focused on the characteristics of the gut microbiota in amphibians, with a particular lack of functional information on how community changes affect microbially mediated functions.

*Rana dybowskii* (brown frog) is an important aquaculture species with both medicinal and nutritional value (Tong et al., 2018). The conditions in which amphibians live in captivity are highly different from those in the wild, and a higher mortality of frogs occurs at higher densities in culture environments (Xiang et al., 2018; Tong et al., 2019). There may be one or more stressors in the cultured environment that indirectly make animals susceptible to disease (Hedrick, 1998; Densmore and Green, 2007). The population of brown frogs bred in captivity has long been characterized by health instability and a high incidence of disease, commonly including diarrhoea (Cui et al., 2007). A variety of factors can cause diarrhoea in amphibians and may be associated with parasitic infections, gastroenteritis and gastrointestinal foreign bodies (Bertelsen and Crawshaw, 2003; Leigh Ann, 2005). Feeding amphibians inappropriate food, such as excessive amounts of simple carbohydrates, can also cause diarrhoea (Clayton, 2005). Brown frog diarrhoea primarily occurs in summer or during the rainy season on unclean farms (Cui et al., 2007). During high-density intensive culture, it is possible to conduct research on the frog gut microbiota and understand the changes in processes and structural differences of this community in frogs at different physiological states, which may provide new ideas for disease surveillance and early warning in amphibians (Jiménez and Sommer, 2017). Despite its importance, the characteristics of the gut microbiota in diseased amphibians have been poorly studied (Rebollar and Harris, 2019).

Amphibians are distinctive among animals medically, morphologically, and physiologically (Densmore and Green, 2007). Their collectively unique life histories and the considerable gaps in our knowledge concerning amphibian diseases and veterinary care increase the difficulty of successfully diagnosing, treating, and maintaining amphibians (Densmore and Green, 2007). An ultimate goal of microbial ecology projects is to predict, assess, and add host health status based on the gut microbiota assembly (Xiong et al., 2017). In the present study, samples of healthy and diarrhoeic brown frogs were collected, Illumina sequencing techniques were used to analyze the response mechanism of the gut microbiota in diseased and healthy frogs, and functional predictions were performed using PICRUSt to test the following hypotheses: (1) significant differences occur in the gut microbiota diversity of healthy and diarrhoeic brown frogs; (2) screening for sensitive microbial populations can indicate the health of brown frogs; and (3) changes in the gut bacterial community result in changes in function, i.e., there is no functional redundancy of the intestinal microorganisms of the brown frog.

## Materials and Methods

### Sample Collection

The healthy and diarrhoeic brown frogs used in this study were taken from three different farms in Jixi City, Heilongjiang Province, China. The conditions of the farms were basically similar. The frogs of the farms were fed daily live yellow mealworm larvae (*Tenebrio molitor*) and house fly larvae (*Musca domestica* L.). The ratio of yellow mealworm larvae to house fly larvae in each farm is different, and the feed of fly larvae in each farm is also different. Fly larvae should be fasted for more than 24 h before feeding frogs, and larvae should be cleaned before feeding frogs. At the end of May, approximately 3,200 brown frogs were stocked in each enclosure at a density of approximately 50/m^2^. At the time of sampling, one of the farms was in the midst of a disease outbreak, with a daily death toll of 200–300 per enclosure. We sampled three separate groups of brown frogs: one group representing frogs with diarrhoea (the DS group) and two groups representing healthy frogs (the HA and HB groups). The DS group (6 samples, DS1–DS6) was sampled on July 05, 2017, and the male-to-female ratio was 3:3. The HA group (5 samples, HA1–HA5) was sampled on July 15, 2017, and the male-to-female ratio was 2:3. The HB group (7 samples, HB1–HB7) was sampled on September 15, 2017, and the male-to-female ratio was 3:4. The body mass of the frogs were 20.51 ± 1.25 g in the CY group, 21.12 ± 2.01 g in the HA group and 22.36 ± 2.65 g in the HB group.

Brown frogs are completely terrestrial frogs, and they are mostly found in dense vegetation during the summer months, thus increasing the difficulty of observing their defecation (Beard et al., 2002). However, compared to the feces of other frogs living in water, such as bullfrogs and frogs, the feces of brown frogs are discharged on land, making the feces content, shape and composition easier to observe (Cui et al., 2007). The feces of brown frogs varies according to the individual (length or body mass) and has a gray-black color, and the feces length of 2-year-old brown frogs is approximately 1.0 cm (Cui et al., 2007).

This study observed that the number of defecations of brown frogs with diarrhoea significantly exceeded the normal frequency. The manure was striped and sticky, had a high water content and included undigested food (such as all or part of the house fly larvae), pus and mucus. The symptoms of diarrhoea in brown frog are very different from those in other animals (such as mammals) (Gomez et al., 2017). Diseased brown frogs may fast after disease onset, and brown frogs can absorb water through the skin and thus do not need to drink water, which shortens the duration of the diarrhoea.

The gut contents were sampled from frog intestines within 20 min after euthanasia. Euthanasia was performed as follows: a glass dryer was laid with gauze, and then each frog was anesthetized by placing a cotton ball immersed with an ether and alcohol mixture underneath (Tong et al., 2020). After flexing of the frog neck, the foramen magnum was observed and a firm metal rod was inserted and rotated cranially to break the distant brain and spinal cord. Prior to disposing of the euthanized amphibians, frog death was confirmed by physical euthanasia or by detecting the stop of the heartbeat. The gut was cautiously isolated from the body, and a portion beginning after the stomach (no stomach) and extending to the anus was collected. A new pair of sterile tweezers was used at each sampling time to prevent cross-pollution. Each sample was put into a sterile vial and rapidly maintained at −80°C.

### DNA Extraction and PCR Amplification

After sample homogenization, DNA from gut microbes was extracted using a FastDNA^®^ spin kit for soil (MP Biomedical, United States) according to the manufacturer’s instructions. The DNA quality was detected by 1% agarose gel electrophoresis, and the DNA quantity and A260/A280 ratio were measured on a NanoDrop 2000 spectrophotometer (Thermo Scientific, United States). Then, 16S rRNA genes in V3–V4 were amplified with primers 806R (5′-GGACTACHVGGGTWTCTAAT-3′) and 338F (5′-ACTCCTACGGGAGGCAG CAG-3′) under the following conditions: 95°C for 3 min; 27 cycles of 95°C for 30 s, 55°C for 30 s, and 72°C for 45 s; and 72°C for 10 min. The solutions for PCR amplification were 20 μl each, including 5 × FastPfu buffer (4 μl), FastPfu polymerase (0.4 μl), 2.5 mM dNTPs (2 μl), each primer (5 μM and 0.8 μl), template DNA (10 ng) and sterilized ddH_2_O. The PCR products were isolated from a 2% agarose gel and treated on an AxyPrep DNA gel extraction kit (Axygen Biosciences, United States). The DNA quantity was assessed with QuantiFluor^TM^-ST (Promega, United States).

### Illumina MiSeq Sequencing

The amplicon levels were standardized, and the samples were gathered. Then, library QC, quantitation, and paired-end sequencing (2 × 300) were performed on a MiSeq system (Illumina, United States). The raw reads were submitted to the NCBI Sequence Read Archive (ID: SUB140047, SUB6512769, and SUB3350790).

### Processing of Sequencing Data

The obtained data were processed with UCHIME (version 1.17) (Edgar et al., 2011) and converted to fastq files. Paired-end sequences were merged using FLASH when the overlapping sequence was longer than 10 bp, and quality filtering was performed using mothur (Whiles et al., 2006) to discard any sequence with homopolymers of 6 bp and blur bases. Chimeras were removed using UCHIME (Edgar et al., 2011). After subsampling each sample to an equal sequencing depth and clustering, 886 operational taxonomic units (OTUs) at 97% identity were obtained, with the number of OTUs ranging from 137 to 472 per sample.

### Ecological and Statistical Analyses

The alpha diversities [phylogenetic diversity (Pd), observed richness (Sobs), and Shannon indices] of the gut microbiotas between diarrhoeic frogs and healthy frogs were calculated using mothur^1^ (Schloss et al., 2011). For continuous variables (Pd, Sobs, and Shannon indices), we used the Shapiro–Wilk (SW) test to assess whether the data conformed to a normal distribution. When the data met the normal distribution, the Levene test was used to test whether the variances were equal. If the variances were equal, then an analysis of variance (ANOVA) was used. Tukey’s test was used to perform a pairwise comparison of significant differences, while the Kruskal–Wallis test was used if the variance was not equal or did not obey the normal distribution. When the Kruskal–Wallis rank-sum test showed a significant difference, the Nemenyi test was used for comparisons. All statistical analyses were performed in the R software environment (version: 3.6.3). A value of *P* < 0.05 was considered significant.

Differences in the bacterial communities between groups were comparatively analyzed by computing the Bray–Curtis dissimilarity and weighted UniFrac similarity from an OTU-level table (Küng et al., 2014) and by non-metric multidimensional scaling (NMDS) for visualization. The diversity of communities at the sequencing depth of each sample was determined from rarefaction curves. Relative abundance was compared between groups of bacterial taxa using the Wilcoxon rank-sum test. The significance level was *P* < 0.05.

Unique and shared genera were identified from the Venn diagrams plotted in R package 3.1.0 (R Core Team, New Zealand), and the core OTUs among all samples and representing ≥0.1% of the reads were assigned. The chi-square test and Fisher’s exact test were used to assess differences in the unique and shared microbial taxa. The differences at the phylum and genus levels were recalculated, and the relative abundance (<0.01% of OTUs in each sample) was analyzed between groups via the Wilcoxon rank-sum test and multiple test correction (Benjamini–Hochberg FDR). The corrected *P* level was <0.05. We used the linear discriminant analysis (LDA) effect size (LEfSe) to identify significant associations between bacterial taxa and host groups (Segata et al., 2011). This index, which accounts for both bioconsistency and significance, was examined to identify differentially abundant OTUs between the control and diarrhoeic animals (DS vs. HA and DS vs. HB).

The functional shifts in the microbiotas of different groups (DS vs. HA and DS vs. HB) were predicted using Phylogenetic Investigation of Communities by Reconstruction of Unobserved States (PICRUSt) (Kanehisa et al., 2012), which can predict the Kyoto Encyclopaedia of Genes and Genomes (KEGG) Ortholog (KO) functional profiles of microbial communities via 16S rRNA gene sequences (Langille et al., 2013) and link OTUs with gene content via a phylogenetic tree of 16S rRNA gene sequences. Thus, such predictions depend on the tree structure and recognition of the closest neighbor, even for large spaces. The relative abundance variations between groups were contrasted via the rank-sum test. *P* < 0.05 was considered significant.

The overall differences in phylogenetic composition and predicted functional compositions were evaluated through a principal coordinate analysis and analysis of similarity using the Bray–Curtis distance, and the association between the changes in the compositions was tested through Pearson correlation based on Mantel tests. The indicator taxa linked with each group were recognized by the IndVal (indicator values) (Dufrêne and Legendre, 1997). Rare taxa were discarded since rare taxa will mistakenly imply special taxa (Pandit et al., 2009). Only taxa with relative abundances >0.1% and significant IndVal values > 0.95 (*P* < 0.05) were chosen (Dufrêne and Legendre, 1997; Logares et al., 2013). The analytical tool was the “*labdsv*” package in R v3.0.0.

## Results

### Alpha Diversity of Gut Microbiota and Shared Microbiota

The sequences were grouped as OTUs at >97% identity, and 886 OTUs that were 443 bp long per read on average were acquired. The samples contained 287.11 ± 113.14 OTUs, varying from 137 (HB6) to 472 (DS5). In the rarefaction tests, the majority of sequenced samples, especially the samples in the HB group, arrived at the plateau stage (Supplementary Figure S1).

**FIGURE 1:**
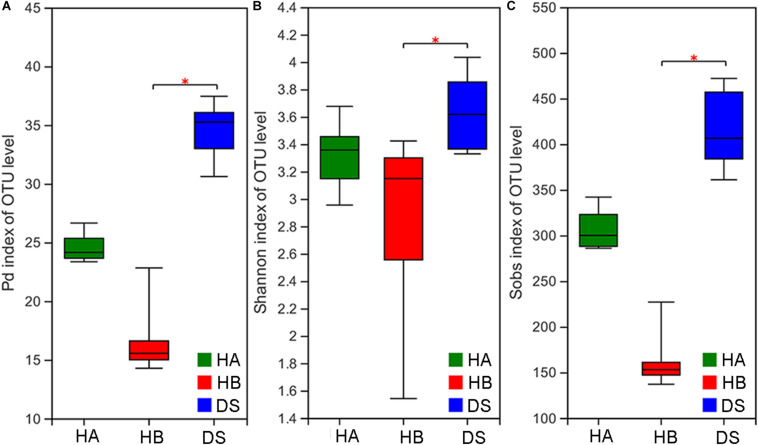
Comparison of alpha diversity in the gut microbiota of diarrhoeic and healthy groups. Comparison of the phylogenetic diversity index **(A)**, Shannon index **(B)**, and Sob index **(C)** of the gut microbiota between the HA or HB group and DS group (*0.01 < *P* ≤ 0.05).

The *P*_*d*_, Shannon, and Sobs indices were significantly different among the three groups (Kruskal–Wallis rank sum test, *P* < 0.001; ANOVA, *P* = 0.028; Kruskal–Wallis rank sum test, *P* < 0.001). The *P*_*d*_, Shannon, and Sobs indices in the DS group were significantly different than those determined for the DS and HB groups (Nemenyi test, *P* < 0.001; Tukey test, *P* = 0.024; Nemenyi test, *P* < 0.001; Figure 1), whereas no difference was observed between the DS and HA groups (Nemenyi test, *P* > 0.05; Tukey test, *P* > 0.05; Nemenyi test, *P* > 0.05; Figure 1).

**FIGURE 2:**
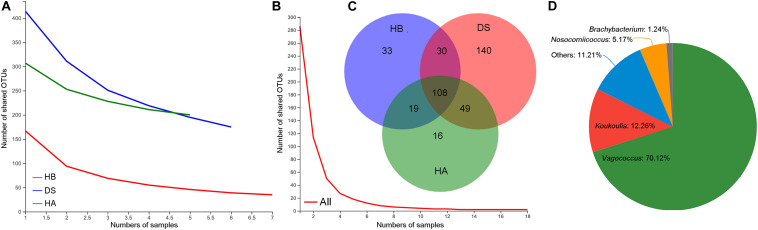
Shared and unique microbiota. Core OTUs of the HA, HB, and DS groups **(A)**, core OTUs of all frogs **(B)**, shared and unique genera among healthy (HA and HB groups) and diarrhoeic (DS group) brown frogs **(C)**, and unique genera in diarrhoeic brown frogs (DS group) **(D)**.

As the number of samples increased, the number of core OTUs in the DS and HA groups slightly decreased while the number of core OTUs in the HB group and in all frogs decreased to a greater extent (Figures 2A,B). The core OTU numbers in the HA, HB, and DS groups and all frogs were 200, 175, 75, and 2, respectively (Figures 2A,B). The core OTUs among all frogs were OTU655 (Firmicutes, *Erysipelatoclostridium*) and OTU398 (Proteobacteria, *Pseudomonas*). The number of shared bacterial genera in the three groups was 108 (Figure 2C). Significant differences were observed between the DS and HA groups (170/459 vs. 35/389) and the DS and HB groups (189/440 vs. 52/322) in the number of unique microbiota and total microbial components at the genera level (Fisher’s exact test, *P* < 0.001). Venn diagrams revealed that there were many more unique genera in the DS group than the HA and HB groups (Figure 2C). The unique genera in the DS group were primarily *Vagococcus*, *Koukoulia*, *Nosocomiicoccus*, and *Brachybacterium* (Figure 2D).

### Beta Diversity of the Gut Microbiota

**FIGURE 3:**
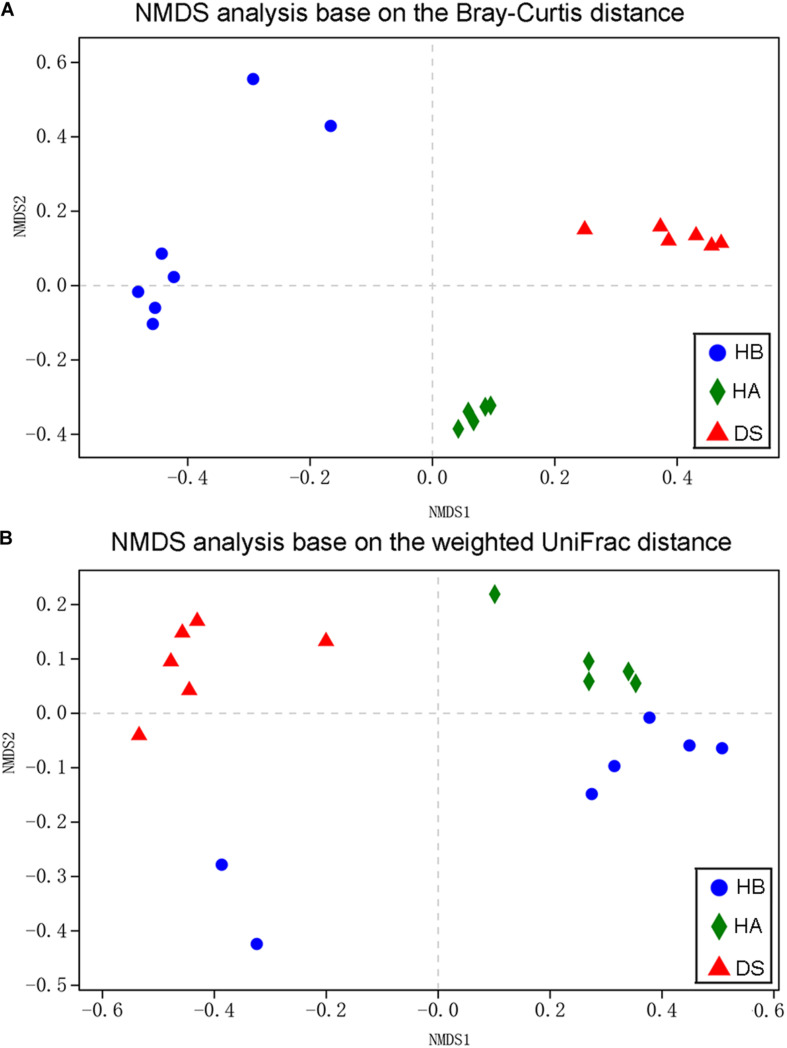
Bacterial community variation among brown frogs of diarrhoeic and healthy groups. Non-metric multidimensional scaling based on all OTUs shows patterns of division by health status (red: DS; green: HA; blue: HB) based on the Bray–Curtis **(A)** and weighted UniFrac **(B)** distance. Each point represents the microbial community of a brown frog in a given group.

Non-metric multidimensional scaling was used to examine the community compositions of the gut microbiota of the three groups (Figure 3). A NMDS analysis based on the Bray–Curtis distance matrix showed that significant separation occurred between the DS and HA groups and the DS and HB groups (Figure 3A). A NMDS analysis based on the weighted UniFrac distance matrix showed that samples from the HA and HB groups were close together while those from the DS and HB groups were significantly separated (Figure 3B). The gut bacterial community compositions differed significantly between the DS and HA groups (ANOSIM: Bray–Curtis, *r* = 1, *P* = 0.005; weighted UniFrac, *r* = 1, *P* = 0.004), and the DS and HB groups (ANOSIM: Bray–Curtis, *r* = 0.8954, *P* = 0.002; weighted UniFrac, *r* = 0.659, *P* = 0.005). Based on the Bray–Curtis distance, the gut bacterial community compositions differed significantly between the HA and HB groups (ANOSIM: Bray–Curtis, *r* = 0.814, *P* = 0.002; Figure 3A); however, based on the weighted UniFrac distance, the compositions were not significant (ANOSIM: weighted UniFrac, *r* = 0.196, *P* = 0.082; Figure 3B).

### Composition of and Variation in Frog Gut Microbiotas

**FIGURE 4:**
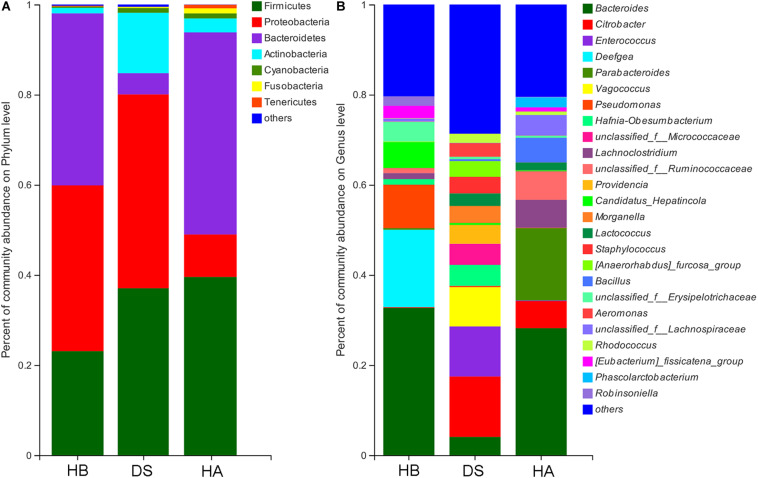
Community bar plot analysis of bacteria at the phylum **(A)** and genus **(B)** levels. Only genera with relative abundances over 2% in at least one sample are shown here.

The dominant phyla (> 1%) in the gut microbiota were Proteobacteria, Firmicutes, Actinobacteria, Bacteroidetes, and Cyanobacteria in the DS group; Bacteroidetes, Firmicutes, Proteobacteria, Actinobacteria, Fusobacteria, and Cyanobacteria in the HA group; and Bacteroidetes, Proteobacteria, Firmicutes, and Actinobacteria in the HB group (Figure 4A and Supplementary Figure S2A). Twenty-two phyla were identified in the DS and HA groups, and 7 phyla (Actinobacteria, Bacteroidetes, Chlamydiae, Chloroflexi, Proteobacteria, Saccharibacteria, and Tenericutes) showed significant differences (Wilcoxon rank-sum test and multiple test correction with Benjamini–Hochberg FD, adjusted *P* < 0.05; Supplementary Figure S3A). Twenty-three phyla were identified in the DS and HB groups, and 7 phyla (Actinobacteria, Bacteroidetes, Chlamydiae, Chloroflexi, Deferribacteres, Saccharibacteria, and TM6__Dependentiae) showed significant differences (Wilcoxon rank-sum test and multiple test correction with Benjamini–Hochberg FD, adjusted *P* < 0.05; Supplementary Figure S3B). Fourteen phyla were identified in the HA and HB groups, and 5 phyla (Deferribacteres, Fusobacteria, Saccharibacteria, Tenericutes, and Verrucomicrobia) showed significant differences (Wilcoxon rank-sum test and multiple test correction with Benjamini–Hochberg FD, adjusted *P* < 0.05; Supplementary Figure S3C).

The dominant genera (>5%) in the gut microbiota were *Citrobacter*, *Enterococcus*, and *Vagococcus* in the DS group; *Bacillus*, *Bacteroides*, *Citrobacter*, *Lachnoclostridium*, *Parabacteroides*, and *unclassified_f__Ruminococcaceae* in the HA group; and *Bacteroides*, *Candidatus*, *Hepatincola*, *Deefgea*, and *Pseudomonas* in the HB group (Figure 4B and Supplementary Figure S2B). Of all 362 genera in the DS and HA groups, 176 showed significant differences between the DS and HA groups (Wilcoxon rank-sum test and multiple test correction with Benjamini–Hochberg FD, adjusted *P* < 0.05; Supplementary Table S1). Of all 255 genera in the HA and HB groups, 78 showed significant differences between the HA and HB groups. (Wilcoxon rank-sum test and multiple test correction with Benjamini–Hochberg FD, adjusted *P* < 0.05; Supplementary Table S2). Of all 255 genera in the HA and HB groups, 78 showed significant differences between the DS and HA groups (Wilcoxon rank-sum test and multiple test correction with Benjamini–Hochberg FD, adjusted *P* < 0.05; Supplementary Table S3).

At all taxonomic levels, communities were more diverse in frogs with diarrhoea than in healthy frogs. At the phylum level, LEfSe revealed that Proteobacteria were significantly more abundant in the DS group and that Bacteroidetes were significantly more abundant in the HA and HB groups (LDA > 4, *P* < 0.05; Supplementary Figures S4, S5). At the genus level, LEfSe revealed that *Aeromonas*, *Citrobacter*, *Enterococcus*, *Hafnia-Obesumbacterium*, *Morganella*, *Providencia*, *Vagococcus*, and *Staphylococcus* were significantly enriched in the DS group compared to the HA group, while *Bacillus*, *Bacteroides*, *Lachnoclostridium*, *Parabacteroides*, *Phascolarctobacterium*, *unclassified-f-Ruminococcaceae*, and *unclassified-f-Lachnospiraceae* were significantly enriched in the HA group (LDA > 4, *P* < 0.05; Supplementary Figure S4). Moreover, at the genus level, LEfSe revealed that *Aeromonas*, *Citrobacter*, *Enterococcus*, *Hafnia-Obesumbacterium*, *Morganella*, *Lactococcus*, *Morganella*, *Providencia*, *Vagococcus*, *Staphylococcus*, and *unclassified-f-Micrococcaceae* were significantly enriched in the DS group compared to the RE group, while *Bacteroides*, *Deefgea*, *Eubacterium*, and *Robinsoniella* were significantly enriched in the HB group (LDA > 4, *P* < 0.05; Supplementary Figure S5). At the genus level, LEfSe revealed that *Bacillus*, *Citrobacter*, *Lachnoclostridium*, *Parabacteroides*, *Phascolarctobacterium*, *unclassified-f-Ruminococcaceae*, and *unclassified-f-Lachnospiraceae* were significantly enriched in the HA group compared to the HB group, while *Deefgea*, *Pseudomonas*, and *Robinsoniella* were significantly enriched in the HB group (LDA > 4, *P* < 0.05; Supplementary Figure S6).

### Indicator Taxa of Frog Health Status

**FIGURE 5:**
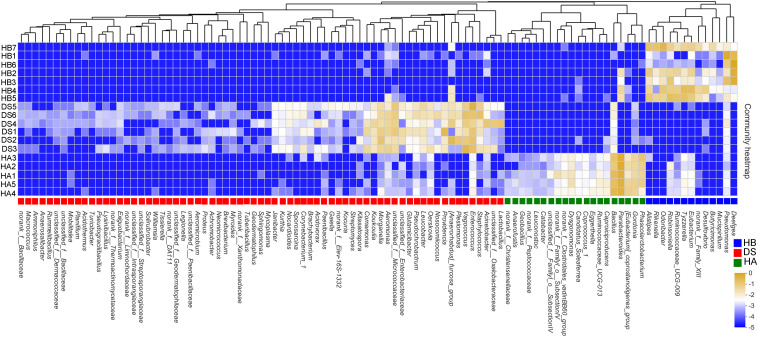
Heatmap showing the relative abundances of the 102 screened indicator taxa between the healthy and diseased groups. The text next to the ordinate presents the name of the sample, and the text under the horizontal information presents the name of the species. The color patch gradient is used to show the abundance changes of different species in the sample. On the right side of the figure, the values represented by the color gradient are shown. Different color patches at the bottom indicate enrichment in this group.

One hundred two sensitive species were selected at the genus level, of which 33 indicated a healthy bacterial status (20 in the HA group and 13 in the HB group) and 69 indicated a diseased bacterial status (e.g., *Acinetobacter*, *Aeromonas*, *Legionella*, *Morganella*, *Proteus*, *Providencia*, *Staphylococcus*, and *Vagococcus*) (Figure 5). A heat map was generated that depicts the normalized abundances of the 102 indicator taxa across the samples and showed their abilities to discriminate among samples according to the sampling site and health status (Figure 5).

### Linear Regression Analysis Between Bacterial Community Structure and Function

**FIGURE 6:**
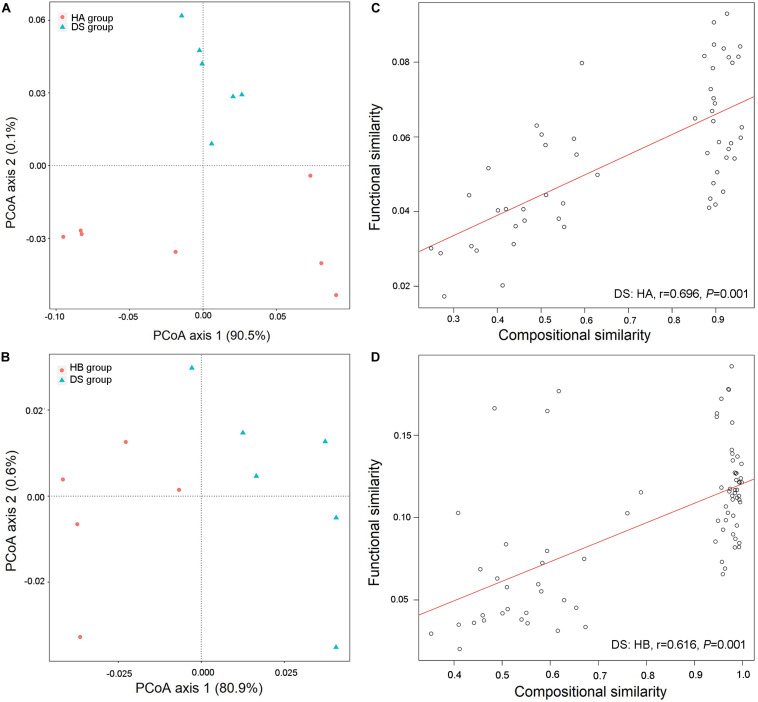
Functional differences in gut microbiota between diarrhoeic and healthy frogs. PCoA of functional structures of bacterial community using the Bray–Curtis distance **(A,B)** and links between compositional and functional similarities **(C,D)**.

Three hundred twenty-six metabolic functional pathways were obtained in the DS and HA groups, and 329 metabolic functional pathways were obtained in the DS and HB groups. The primary coordinate analysis showed significant differences in the functional composition between healthy and diarrhoeic frogs, which was primarily separated by axis 1 (Figures 6A,B). The community similarity test likewise showed a significant difference in functional composition between healthy and diarrhoeic frogs (DS: HA, *R*^2^ = 0.477, *P* = 0.003, Figure 6A; DS: HB, *R*^2^ = 0.223, *P* = 0.047, Figure 6B).

A linear regression analysis showed that the community composition and functional composition of the healthy HA group and the diarrhoeic DS group were significantly and positively correlated (DS: HA: *r* = 0.696, *P* = 0.001, Figure 6C; DS: HB: *r* = 0.616, *P* = 0.001, Figure 6D), indicating that changes in the intestinal bacterial community of brown frogs significantly altered the bacteria-mediated physiological functions.

**FIGURE 7:**
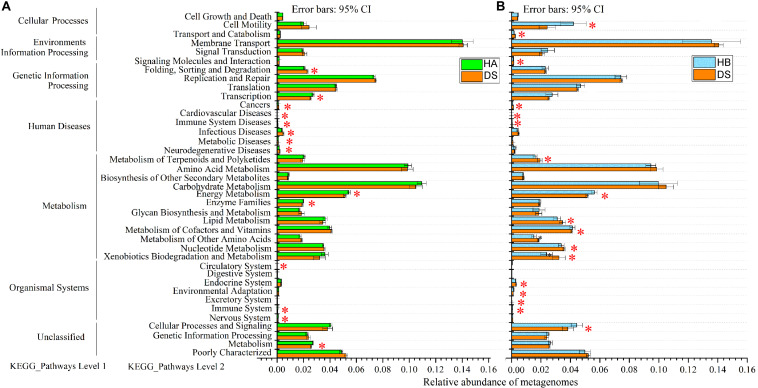
Relative abundance of forecasted genes in the metagenome associated with level-1 and level-2 KEGG pathways. **(A,B)** Red, green, and blue boxes: DS, HA, and HB groups, respectively. The left, middle and right lists represent level-1 and level-2 KEGG pathways and the abundance of each pathway, respectively. Asterisks indicate significant differences among groups.

The predicted genomic functions of the *R. dybowskii* gut microbiota were evaluated using PICRUSt, the results of which are shown in Figure 7. A comparison between the DS and HA groups showed that seven KEGG pathways were enriched in the DS group (immune system diseases, infectious diseases, etc.), and seven pathways were significantly enriched in the HA group (immune system, energy metabolism, etc.) (Figure 7A), while a comparison between the DS and HB groups showed that eleven KEGG pathways (immune system diseases, cardiovascular diseases, etc.) were significantly enriched in the DS group, and six KEGG pathways (immune system, energy metabolism, etc.) were enriched in the HB group (Figure 7B).

## Discussion

In this study, we used Illumina high-throughput sequencing techniques to compare and analyze the gut microbiota composition of diarrhoeic and healthy brown frogs as well as the functional profiles. The results showed significant changes in the composition and function of the gut microbiota associated with *R. dybowskii* disease, and sensitive bacteria genera that indicated the health and disease status of brown frogs were identified. The results of the present study are of great significance for evaluating the health status of brown frogs and elucidating the mechanism of *R. dybowskii* disease occurrence.

### Brown Frog Disease and Management of Frog Culture Health

At present, the brown frog farming industry is stagnant, primarily due to the long growth cycle (at least 18 months from hatching to slaughtering), complex management procedures based on life history (primarily including spawning, hatching, tadpoles, metamorphosis, the growth of 1-year-old young frogs, hibernation, emergence, the growth of 2-year-old young frogs and other stages of feeding and management techniques), and high incidence of disease in frog farms (Tong et al., 2019). Diseases that frequently occur during the growth of frogs are a considerable obstacle to the development of the brown frog aquaculture industry (Xiang et al., 2018; Tong et al., 2019). Most diseases of captive amphibians are directly or indirectly related to feeding and management (Densmore and Green, 2007). Amphibians are notoriously sensitive to captivity (Griffiths and Pavajeau, 2008), and brown frogs may be even more sensitive to conditions on land (Tong et al., 2019). For captive and wild amphibian populations, deviations from ideal environmental conditions can be extremely harmful to health and may be associated with the occurrence and development of disease (Densmore and Green, 2007). Moreover, one or more stressors may occur in the cultured environment that indirectly increase the animals’ susceptibility to disease (Hedrick, 1998; Densmore and Green, 2007).

In brown frog farming, several factors may cause disease (e.g., diarrhoea and red leg syndrome), such as poor breeding management and lack of timely environmental clean-up (inadequate cleaning and disinfection) (Densmore and Green, 2007). Diarrhoeic brown frogs excrete incompletely digested housefly larvae (Cui et al., 2007). Amphibian diarrhoea may be associated with parasitic infections, gastroenteritis, and gastrointestinal foreign bodies (Bertelsen and Crawshaw, 2003; Leigh Ann, 2005). Feeding amphibians inappropriate food, such as excessive amounts of simple carbohydrates, can also cause diarrhoea (Clayton, 2005). Although house fly larvae are easy to breed and inexpensive, they have a high water content and are not easily digested by brown frogs (Wang and Shelomi, 2017). Housefly maggot meal is often used in place of fish meal, which may adversely affect the nutrition and intestinal health of bullfrogs (Li et al., 2019). Live house fly larvae also carry many bacteria inside and outside the body, potentially including many pathogens (Khamesipour et al., 2018). Therefore, the breeding process should be combined with an adjusted feeding structure and the growth of an increased number of yellow powder worms may reduce the occurrence of disease.

### Diversity of the Gut Microbiota of Frogs

The results of the present study showed that the alpha diversity of the DS group (diseased frogs) was higher than that of the HB group, although a significant difference was not observed between the DS and HA groups. Studies have reported significantly reduced alpha diversity (Schoster et al., 2017; Li et al., 2018), no difference in alpha diversity (Gomez et al., 2017; Schoster et al., 2017; Yang et al., 2017), and elevated alpha diversity (Tran et al., 2018) in the gut microbiota after diarrhoea in animals. Even in the absence of disease, microbial alpha diversity can vary widely among animal populations, among individuals within a population, and among different microbiota habitats within the same individual (Huang et al., 2018; Rudi et al., 2018). Thus, the occurrence of disease may be accompanied to some extent by changes in the alpha diversity of gut microbes but not always a significant reduction in alpha diversity.

In the present study, factors that could affect the alpha diversity of the gut microbiota due to diarrhoea may have been complicated by the occurrence of other diseases because some enriched bacterial genera (such as *Aeromonas*, *Morganella*, *Providencia*, *Proteus*, and *Staphylococcus*) are often associated with diarrhoea in animals (Samonis et al., 1997; Cajetan et al., 2010) or other diseases (such as *Aeromonas*, *Citrobacter*, *Proteus*, and *Staphylococcus*), such as red-leg syndrome (Mauel et al., 2002; Pasteris et al., 2006; Schadich and Cole, 2009; Xie et al., 2009; Weng et al., 2016). Bacterial infections may be a common consequence of other problems, such as traumatic injury in unsanitary captive situations, and the pathogens may be secondary invaders following viral infections and mycotic skin infections (Densmore and Green, 2007). These factors may affect the alpha diversity of frog gut microbiota.

In the present study, a significant difference was observed in the beta diversity of the gut microbiota between the diarrhoeic and healthy frogs but not between the HE group and RE group based on weighted UniFrac dissimilarities (Figure 3B). Because the species composition changes with the disease state, some microbiome OTUs may serve as potential diagnostic indicators of disease (Ma et al., 2019). According to the Venn diagram, the proportion of shared genera between the diarrhoeic and healthy frogs was lower and the proportion of unique genera was relatively higher, indicating a lower similarity between the diarrhoeic and healthy groups. Changes in shared species should offer promising diagnostic indicators for animal microbiome-associated diseases (Ma et al., 2019). Therefore, using methods other than bacterial diversity (such as indicator values in the present study) provides us with a great deal of information on host health status as well as a new perspective to diagnose and treat diseases of amphibians (Yu et al., 2018).

In this study, weighted UniFrac results were not significantly different between the HA and HB groups while the Bray–Curtis distance indicated significant differences, which may be related to a number of factors. Different methods of raising house fly larvae may cause differences in the gut microbiota of captive brown frogs. Flies represent the main food for captive-bred frogs; however, the microbiota composition of flies in different farms can vary greatly. House fly larvae are kept in different farms, and the feed used is very different, including discarded human food, cornmeal, and slaughterhouse waste. The composition of the food of captive brown frogs, such as the ratio of house fly larvae to yellow powder larvae, may cause differences in the gut microbiota of these frogs. Differences in culture management, such as the temperature in the greenhouse, may affect the intestinal microbiota of captive brown frogs (Kohl and Yahn, 2016). In this study, we combined the two health groups and compared them with the diseased group, which led to increased differences between the healthy groups and decreased differences between the healthy group and the diseased group; thus, the difference between the groups was difficult to find. In particular, when the sources of health groups differ greatly, this effect will be further amplified.

Significant differences were observed in the composition of the gut microbiota between healthy and diarrhoeic brown frogs. Compared with the gut microbiota composition of healthy brown frogs, the relative abundance of Proteobacteria in diarrhoeic brown frogs increased significantly while the content of Bacteroidetes decreased significantly (Supplementary Figure S3). The relative abundance of Bacteroidetes in the intestines decreased significantly. A reduction in Bacteroidetes is speculated to reduce the feeding vitality of brown frogs (consistent with the observed symptoms of disease), thereby making the frogs more susceptible to pathogenic infection and increasing the risk of disease. In this study, bacteria that are often associated with diarrhoea in animals, namely, *Aeromonas*, *Morganella*, *Providencia*, *Proteus*, and *Staphylococcus* (Samonis et al., 1997; Cajetan et al., 2010), were significantly enriched in the diarrhoeic frogs. We also observed that pathogenic bacteria associated with red-leg syndrome, such as *Aeromonas*, *Citrobacter*, *Proteus*, and *Staphylococcus*, were significantly enriched in the diarrhoeic group (Mauel et al., 2002; Pasteris et al., 2006; Schadich and Cole, 2009; Xie et al., 2009; Weng et al., 2016). Brown frogs with diarrhoea may also be accompanied by the clinical symptoms of red-leg syndrome. Vertebrate intestinal tracts carry a large number of pathogenic microorganisms, and many pathogens do not exhibit pathogenicity under normal conditions but may exhibit strong pathogenicity during gut microbiota dysbiosis (Nagaokitamoto et al., 2016). The occurrence of bacterial diseases may depend on the structure and composition of the gut microbiota or on interactions within the gut microbiota. For example, *Aeromonas* infection is often accompanied by mixed infections (Mosser et al., 2015), suggesting that *Aeromonas* infection may interact with other bacteria and that the resulting disease may depend on the composition of the gut microbiota.

### Biological Sensitivity Was Used to Indicate the Health Status of Frogs

A comparison of the differences in intestinal microbial abundance between healthy and diseased brown frogs revealed that the health status of brown frogs could be indicated and assessed by examining sensitive populations (Yu et al., 2018). We screened 102 bacterial genera in the gut microbiota of healthy and sick brown frogs for indicative microorganisms using indicator values (Figure 5). Known physiological functions and ecological characteristics coincide with the occurrence of disease. For example, *Staphylococcus* can produce the enterotoxins teratosrol and heterottoin, which can cause purulent infections, sepsis and enteritis and may cause death in certain vertebrates (Oladele et al., 2012). *Proteus* products include whiplash, bacillus, endotoxins and hemolytic toxins, which can lead to severe diarrhoea upon infection in animals (Cajetan et al., 2010). *Aeromonas* is the most common pathogenic genus in frog culture and can produce highly dangerous toxins (e.g., hemolytic toxins, tissue toxins, and enterotoxins), and its increased abundance may lead to outbreaks of frog disease (Mosser et al., 2015; Tong et al., 2019). We detected that the relative abundance of *Aeromonas* in the intestines of diseased brown frogs was significantly higher than that in healthy frogs. Therefore, the abundance of potential pathogenic bacteria (such as *Aeromonas* and *Staphylococcus*) in the cultured environment should be controlled to maintain the healthy breeding of brown frogs in the future (Xiang et al., 2018). On the other hand, the relative abundance of *Bacillus* was significantly reduced in the diarrhoeic frogs. *Bacillus* has the ability to suppress harmful bacteria and can produce substances to inhibit the growth of pathogenic bacteria, such as antibiotics, while the production of a variety of digestive enzymes can help the host digest nutrients, thereby resulting in a dual role in disease prevention and control (Elshaghabee et al., 2017). *Bacillus* has been used as a probiotic to improve the health of vertebrates (Hai, 2015). Therefore, the health status of brown frogs can be assessed by detecting the dynamic changes in sensitive bacteria, which can provide a reference for the healthy breeding of brown frogs (Xiong et al., 2017; Yu et al., 2018).

### Differences in the Function of the Gut Microbiota of Healthy and Diarrhoeic Frogs

Because the occurrence of host disease often accompanies the destruction of the dynamic balance of the gut microbiota, the gut microbiota plays an important role in promoting host health (Sánchez et al., 2017). Although this view has become widely accepted, because different microorganisms can perform similar functions, functional redundancy is thought to occur in the microbiota (Moya and Ferrer, 2016). Studies have shown that different species of frogs have different gut microbiota structures but that these bacterial communities have similar biological functions (Vences et al., 2016). Therefore, how changes in the gut microbiota associated with disease affect physiological functions remains unclear (Yu et al., 2018). The results of the present study showed that changes in the gut microbiota of the brown frog were accompanied by significant changes in the predicted functions of the gut microbiota; moreover, a significant positive correlation occurred between the structural similarity and the functional similarity of the bacterial community, indicating that the functional redundancy of the gut microbiota of the brown frog was relatively low (Figure 6). The functional redundancy of microbes in the gut ecosystem may be different in different species of animals (Schwarz et al., 2016). The diverse gut microbiota of mammals presents functional redundancy that may buffer shifts in composition (Lozupone et al., 2012), although some animal species (including some amphibians and insects) typically have a much lower microbiota diversity and consequently may be more affected by dysbiosis (Schwarz et al., 2016). The same species of animals may have different functional redundancy at different developmental stages or during disease progression. For example, redundancy in the infant gut may be much higher than that observed in the adult gut (Moya and Ferrer, 2016). On the other hand, the consequences of different age-, diet-, and disease-induced trajectories are strongly influenced by functional redundancy (Moya and Ferrer, 2016). Changes in the gut microbiota have been associated with disease and intervention, such as antibiotic treatment and diarrhoea (Samonis et al., 1997; Cajetan et al., 2010). Therefore, disease may also be accompanied by a loss of functional redundancy over time, which may be associated with the severity of the dysbiosis and the disease progression (Moya and Ferrer, 2016). Additionally, in the present study, changes in predicted function were consistent with the disease symptoms. For example, in diarrhoeic frogs, the immune system was significantly reduced and disease-related functions were significantly enriched (Figure 7). Therefore, the occurrence of the disease is accompanied by changes in the gut microbiota that reduce the functions associated with digestion, absorption, and energy metabolism, thereby resulting in a decrease in the animal’s feeding capacity and a reduction in the energy involved in the immune response, thereby aggravating *R. dybowskii* disease.

## Conclusion

In the present study, we investigated for the first time the impacts of disease on the gut bacterial community of frogs. The results showed that disease was accompanied by significant changes in the composition and function of the gut microbiota. Bacterial genera responsive to variations in health state and possible indicator taxa were identified, and their dynamic modes were identical with their relevant ecofunctions, thus providing a foundation for the development of gut microbial treatment schemes for frog health control. A significant and positive association was observed between the composition and functional composition of the gut microbiota community, indicating a low functional redundancy of the frog gut bacterial community. The results of the study are important for evaluating the health of brown frogs and elucidating the mechanisms of disease occurrence.

## Data Availability Statement

The raw reads were deposited in the NCBI Sequence Read Archive (SRA) database (Accession Numbers: PRJNA422729, PRJNA587796, and PRJNA587644).

## Ethics Statement

The animal study was reviewed and approved by all applicable international, national, and/or institutional guidelines for the care and use of animals were followed. All procedures followed the Guide for the Care and Use of Laboratory Animals and were reviewed and approved by the IACUC of Northeast Agricultural University (IACUC#2015-035). This article does not contain any studies with human participants performed by any of the authors.

## Author Contributions

QT, Z-FH, and L-YC: data collection, data analysis, and interpretation and drafting of the article. J-TZ, H-BW, and QT: conception or design of the work. QT, L-YC, JB, and J-HX: sample collection. QT, J-TZ, and Z-FH: writing and critical revision of the article. J-TZ: final approval of the version submitted. All authors contributed to the article and approved the submitted version.

## Conflict of Interest

The authors declare that the research was conducted in the absence of any commercial or financial relationships that could be construed as a potential conflict of interest.

## Footnotes

1 www.mothur.org/
