# Supplementary material for: Comparison of Gut Microbiota Diversity and Predicted Functions Between Healthy and Diseased Captive Rana dybowskii
Source: Front Microbiol. 2020 Sep 1;11:2096. doi: 10.3389/fmicb.2020.02096 (PMC7490342; doi:10.3389/fmicb.2020.02096)
Supplement: Supplementary file 1 [file Data_Sheet_1.docx]

**Comparison of gut microbiota composition and predicted functions between healthy and diseased captive *Rana dybowskii***

Qing Tong^1,2^, Li-yong Cui^2^, xiao-peng Du^1^, Zong-fu Hu^1^, Jia Bie^1^, Hong-bin Wang^1^, Jian-tao Zhang1^*^

1 College of Veterinary Medicine, Northeast Agricultural University, Harbin 150030, China;

2 Jiamusi Branch of Heilongjiang Academy of Forestry Sciences, Jiamusi, 154000, China.

*Corresponding author. Mailing address: College of Veterinary Medicine, Northeast Agricultural University, Harbin, 150030, People’s Republic of China.

Tel.: +86-451-55190470. Fax: +86-451-55190470.

1. mail: zhangjiantao@neau.edu.cn.


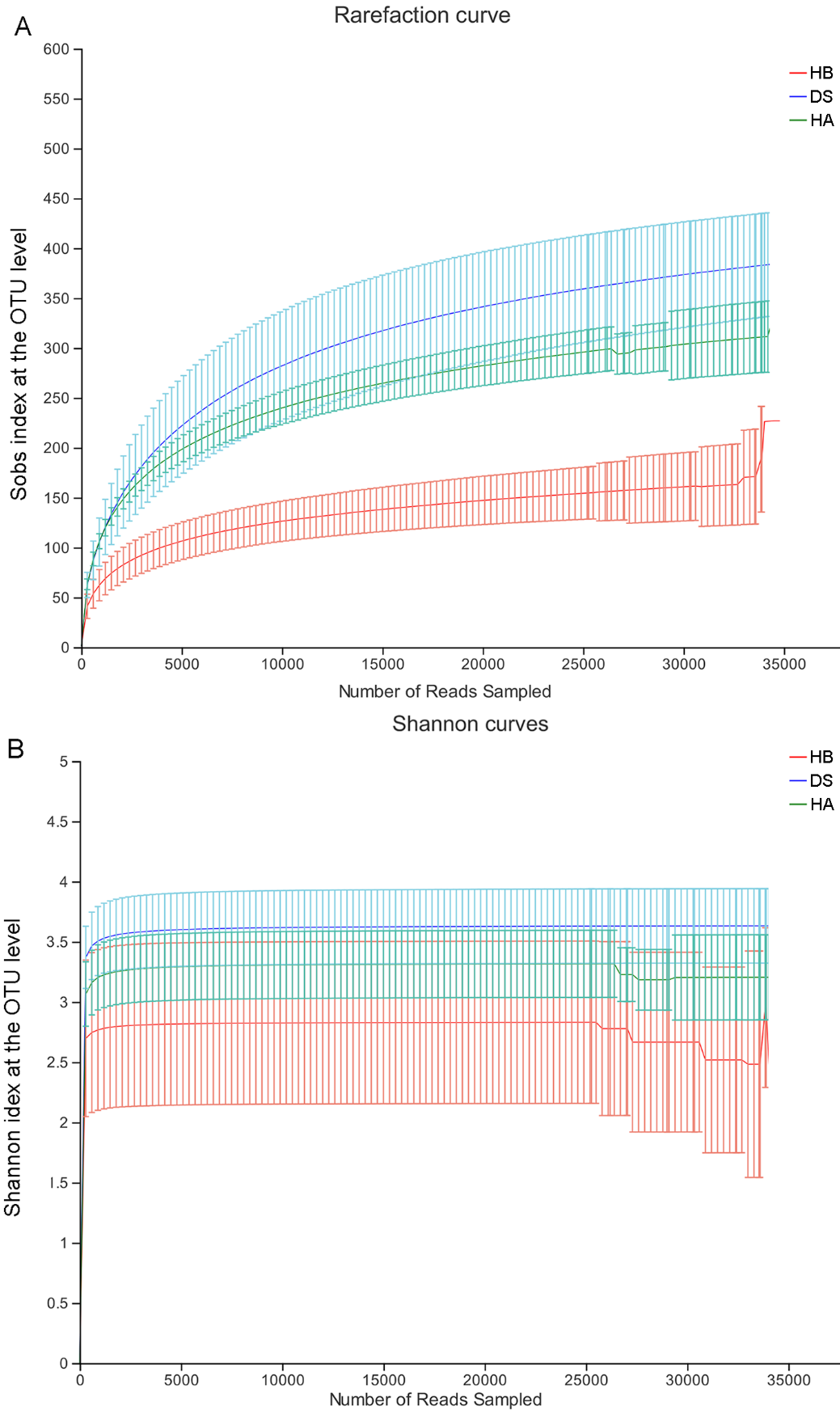


**Figure S1** Rarefaction curves (A) and Shannon curves (B).


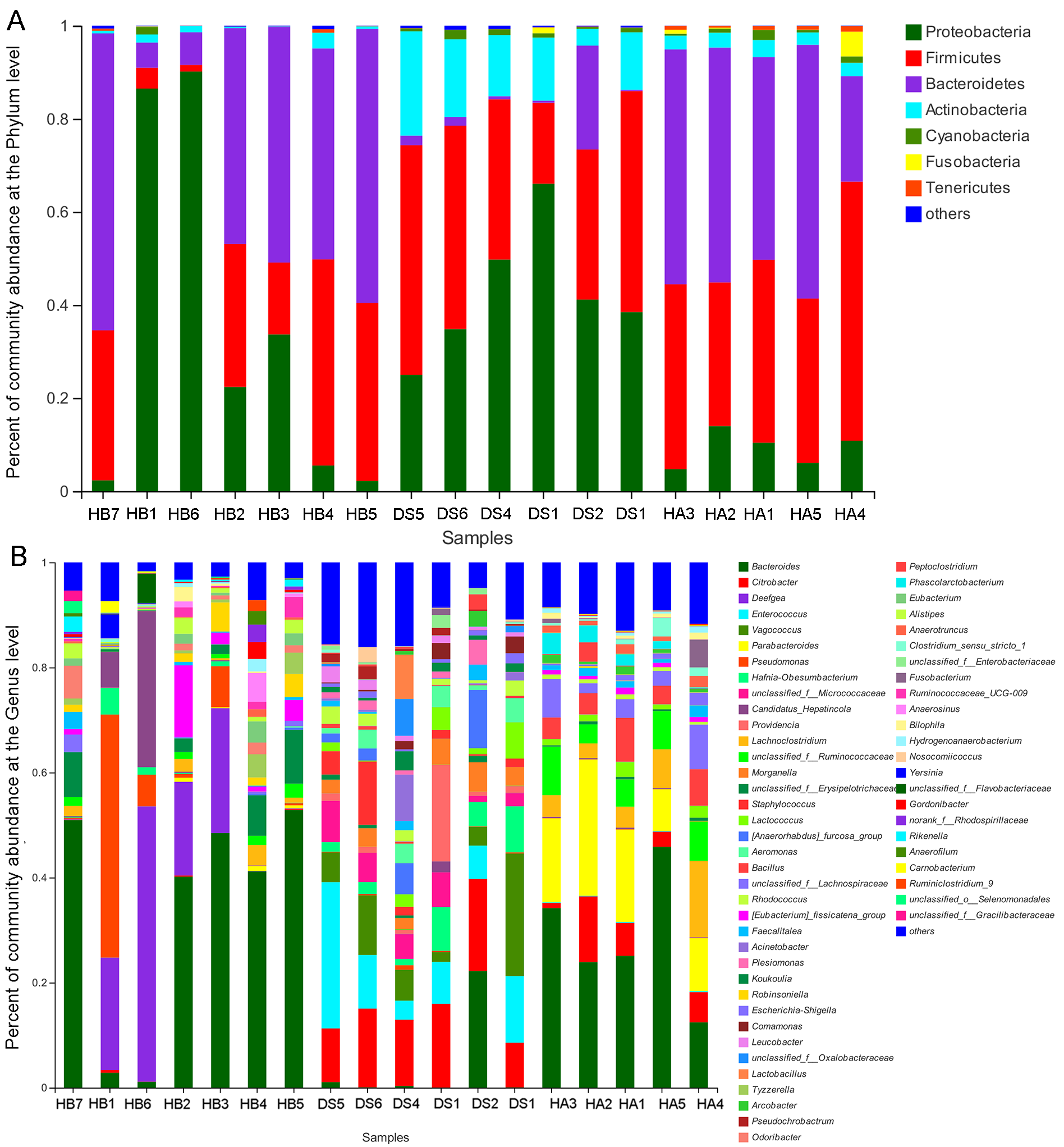


**Figure S2** Community bar plot analysis of bacteria at the phylum (A) and genus (B) levels.

Only phyla with relative abundances over 1% in at least one sample are shown here. Only genera with relative abundances over 2% in at least one sample are shown here.


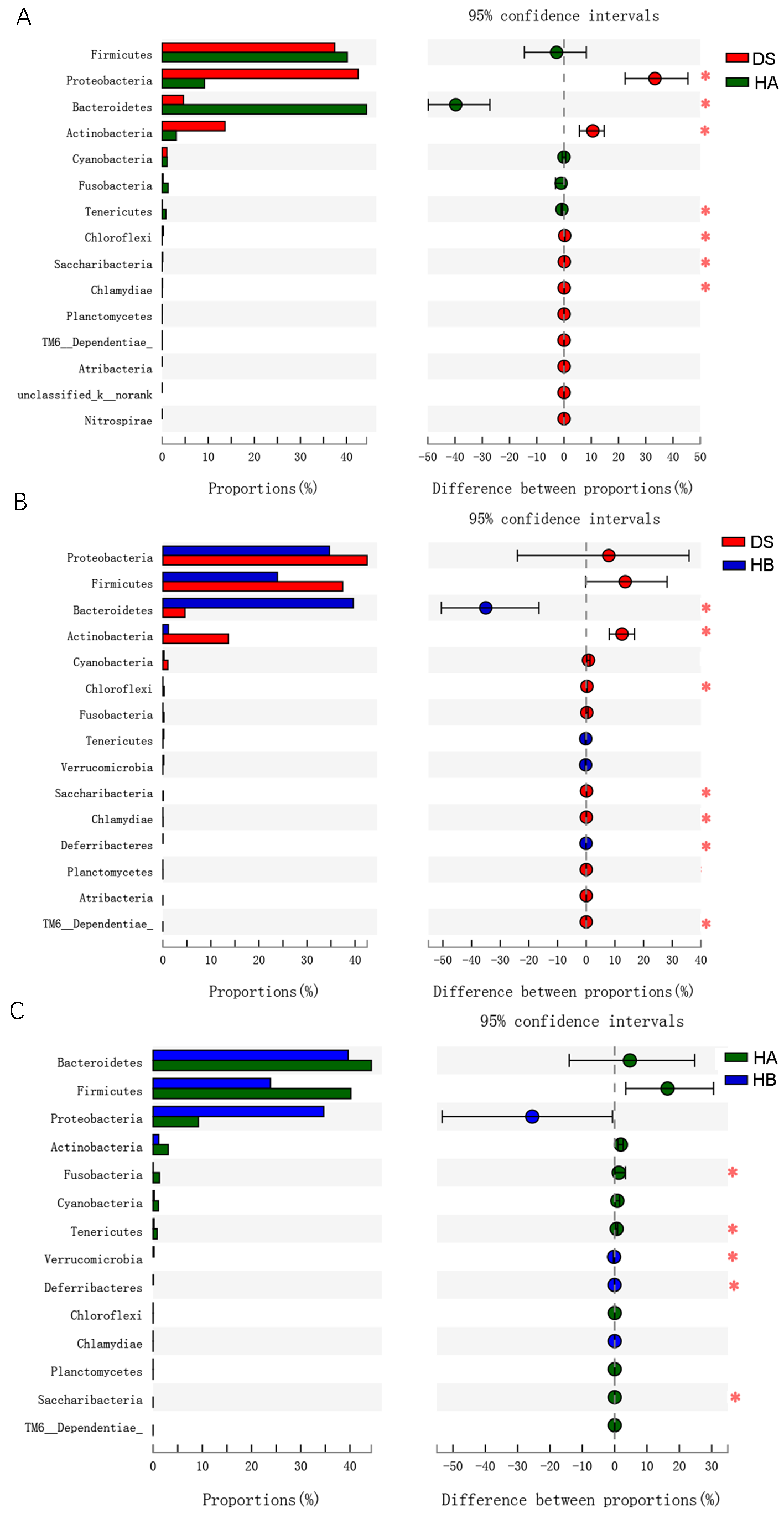


**Figure S3** Differences in the relative abundance of major bacterial phyla between the DS and HA groups (A), DS and HB groups (B), and the HA and HB groups (C). The significance of major bacterial phyla was determined using the Wilcoxon rank-sum test. Asterisks indicate significant differences among groups.


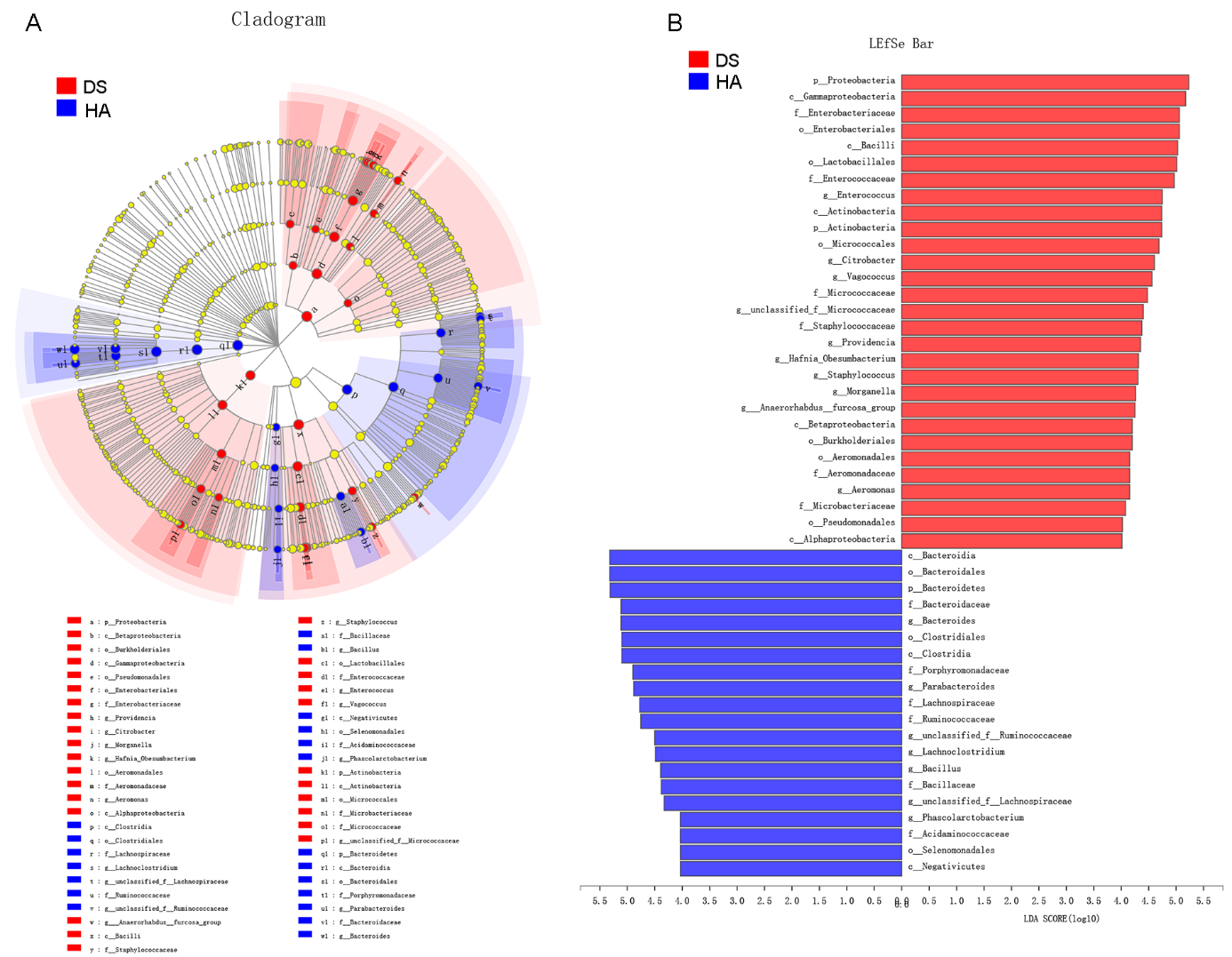


**Figure S4** Cladogram of the phylogenetic tree of microbial lineages related to differences in gut groups using LEfSe.

Differences in the CY and HE groups are represented by treatment colour (red: DS samples, blue: HA samples). Circle diameter is proportional to taxon abundance. The multiclass analysis is non-strict (at least one class is differential). Circles from in-to-out and from out-to-in indicate the taxonomic ranks from domain to genus and from phylum to genus, respectively. The scores with LDA > 4 are displayed for all the taxa.


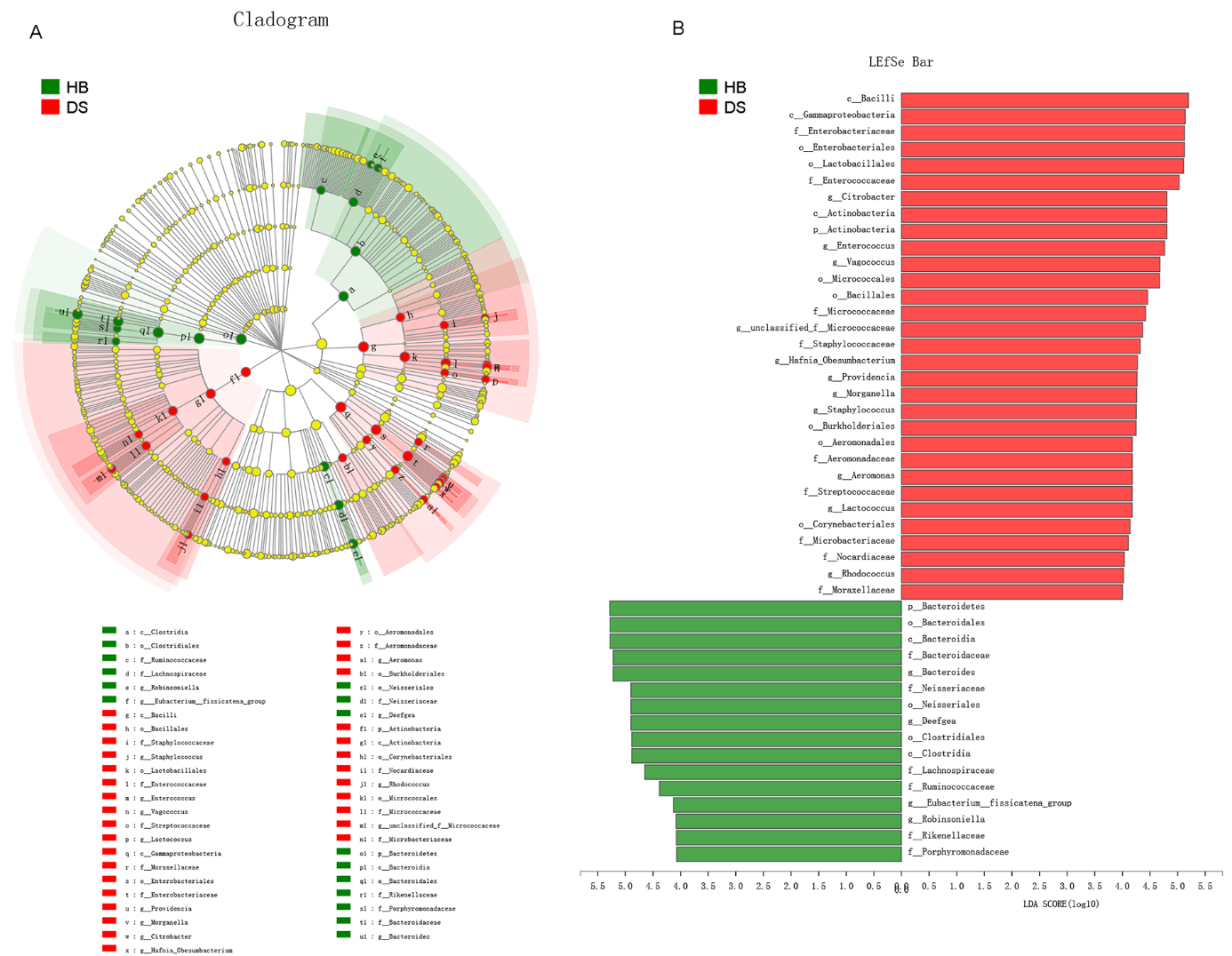


**Figure S5** Cladogram of the phylogenetic tree of microbial lineages related to differences in gut groups using LEfSe.

Differences in the DS and HB groups are represented by treatment colour (red: DS samples, green: HB samples). Circle diameter is proportional to taxon abundance. The multiclass analysis is non-strict (at least one class is differential). Circles from in-to-out and from out-to-in indicate the taxonomic ranks from domain to genus and from phylum to genus, respectively. The scores with LDA > 4 are displayed for all the taxa.


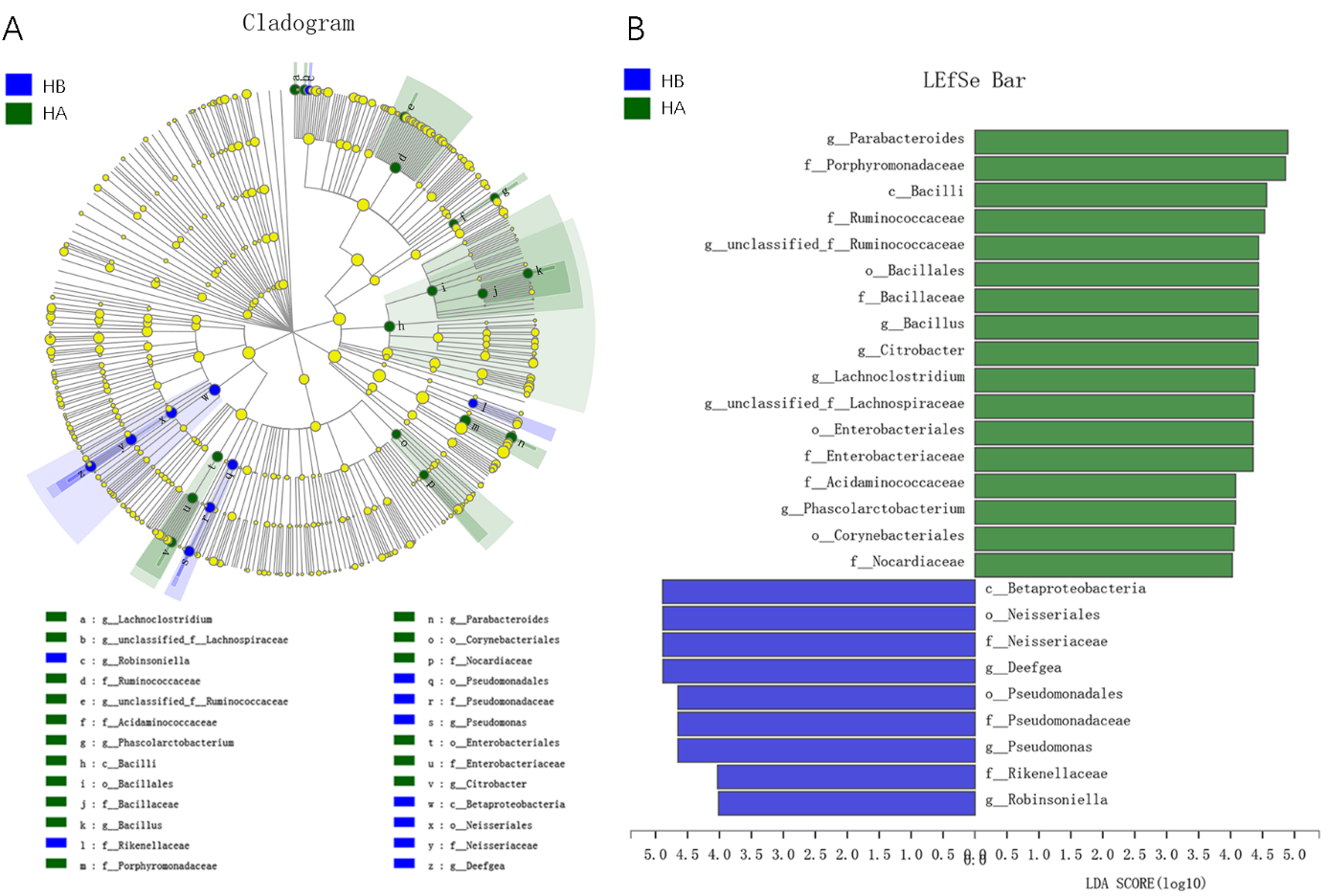


**Figure S6** Cladogram of the phylogenetic tree of microbial lineages related to differences in gut groups using LEfSe.

Differences in the HA and HB groups are represented by treatment colour (green: HA samples, blue: HB samples). Circle diameter is proportional to taxon abundance. The multiclass analysis is non-strict (at least one class is differential). Circles from in-to-out and from out-to-in indicate the taxonomic ranks from domain to genus and from phylum to genus, respectively. The scores with LDA > 4 are displayed for all the taxa.

**Table S1** Differences between the DS and HA groups at the genus level (Wilcoxon rank-sum test, *P* < 0.05).

| Species name | DS-Mean (%) | DS-Sd (%) | HA-Mean (%) | HA-Sd (%) | *P* value | Corrected *p* value | Lower ci | Upper ci | Effectsize |
| --- | --- | --- | --- | --- | --- | --- | --- | --- | --- |
| *g__Bacteroides* | 3.961 | 8.948 | 28.300 | 12.490 | 0.014 | 0.036 | -36.590 | -13.010 | -24.340 |
| *g__Citrobacter* | 13.300 | 3.482 | 5.690 | 4.385 | 0.022 | 0.046 | 3.113 | 11.540 | 7.607 |
| *g__Parabacteroides* | 0.000 | 0.001 | 15.520 | 7.079 | 0.005 | 0.025 | -21.110 | -9.988 | -15.520 |
| *g__Enterococcus* | 11.450 | 8.619 | 0.129 | 0.047 | 0.008 | 0.025 | 6.207 | 18.580 | 11.320 |
| *g__Vagococcus* | 8.616 | 7.891 | 0.000 | 0.000 | 0.005 | 0.025 | 3.743 | 15.140 | 8.616 |
| *g__unclassified_f__Ruminococcaceae* | 0.037 | 0.022 | 6.551 | 2.179 | 0.008 | 0.025 | -8.108 | -4.710 | -6.514 |
| *g__Lachnoclostridium* | 0.004 | 0.005 | 6.572 | 4.770 | 0.008 | 0.025 | -10.760 | -3.538 | -6.568 |
| *g__Bacillus* | 0.418 | 0.267 | 5.393 | 2.136 | 0.008 | 0.025 | -6.765 | -3.424 | -4.975 |
| *g__unclassified_f__Lachnospiraceae* | 0.068 | 0.095 | 4.825 | 2.913 | 0.008 | 0.025 | -7.083 | -2.456 | -4.757 |
| *g__unclassified_f__Micrococcaceae* | 4.724 | 2.539 | 0.016 | 0.006 | 0.008 | 0.025 | 2.754 | 6.558 | 4.708 |
| *g__Hafnia-Obesumbacterium* | 4.507 | 3.340 | 0.001 | 0.002 | 0.008 | 0.025 | 2.162 | 6.850 | 4.506 |
| *g__Providencia* | 3.965 | 7.067 | 0.002 | 0.003 | 0.008 | 0.025 | 0.930 | 9.771 | 3.963 |
| *g__Morganella* | 3.712 | 1.334 | 0.038 | 0.056 | 0.008 | 0.025 | 2.723 | 4.752 | 3.674 |
| *g__Staphylococcus* | 3.621 | 4.338 | 0.005 | 0.005 | 0.008 | 0.025 | 1.191 | 7.293 | 3.616 |
| *g__[Anaerorhabdus]_furcosa_group* | 3.518 | 4.306 | 0.008 | 0.008 | 0.022 | 0.046 | 0.684 | 6.601 | 3.510 |
| *g__Aeromonas* | 2.971 | 1.599 | 0.039 | 0.027 | 0.008 | 0.025 | 1.792 | 4.046 | 2.933 |
| *g__Phascolarctobacterium* | 0.001 | 0.002 | 2.190 | 1.480 | 0.005 | 0.025 | -3.297 | -1.048 | -2.189 |
| *g__Acinetobacter* | 1.896 | 3.433 | 0.029 | 0.018 | 0.008 | 0.025 | 0.173 | 4.722 | 1.867 |
| *g__Plesiomonas* | 1.616 | 1.609 | 0.000 | 0.000 | 0.005 | 0.025 | 0.660 | 2.874 | 1.616 |
| *g__Koukoulia* | 1.550 | 1.120 | 0.000 | 0.000 | 0.005 | 0.025 | 0.862 | 2.461 | 1.550 |
| *g__unclassified_f__Oxalobacteraceae* | 1.475 | 2.750 | 0.001 | 0.002 | 0.007 | 0.025 | 0.112 | 3.727 | 1.474 |
| *g__Leucobacter* | 1.455 | 0.905 | 0.014 | 0.005 | 0.008 | 0.025 | 0.856 | 2.162 | 1.441 |
| *g__Gordonia* | 0.031 | 0.028 | 1.417 | 0.189 | 0.008 | 0.025 | -1.523 | -1.228 | -1.386 |
| *g__Anaerotruncus* | 0.012 | 0.012 | 1.396 | 0.579 | 0.008 | 0.025 | -1.839 | -0.932 | -1.384 |
| *g__Comamonas* | 1.395 | 1.445 | 0.007 | 0.005 | 0.008 | 0.025 | 0.387 | 2.388 | 1.388 |
| *g__Microbacterium* | 0.950 | 0.477 | 0.212 | 0.097 | 0.014 | 0.036 | 0.364 | 1.087 | 0.739 |
| *g__Pseudochrobactrum* | 1.065 | 0.866 | 0.051 | 0.027 | 0.008 | 0.025 | 0.426 | 1.716 | 1.014 |
| *g__[Eubacterium]_coprostanoligenes_group* | 0.000 | 0.001 | 1.037 | 0.464 | 0.005 | 0.025 | -1.399 | -0.674 | -1.037 |
| *g__Oerskovia* | 0.940 | 0.791 | 0.054 | 0.024 | 0.014 | 0.036 | 0.321 | 1.440 | 0.886 |
| *g__[Eubacterium]_fissicatena_group* | 0.003 | 0.004 | 0.919 | 0.238 | 0.008 | 0.025 | -1.116 | -0.752 | -0.916 |
| *g__Ruminiclostridium_5* | 0.019 | 0.047 | 0.878 | 0.280 | 0.007 | 0.025 | -1.102 | -0.654 | -0.858 |
| *g__norank_o__Mollicutes_RF9* | 0.000 | 0.000 | 0.818 | 0.290 | 0.004 | 0.025 | -1.066 | -0.577 | -0.818 |
| *g__Bilophila* | 0.000 | 0.000 | 0.785 | 0.438 | 0.004 | 0.025 | -1.115 | -0.428 | -0.786 |
| *g__Glutamicibacter* | 0.736 | 0.472 | 0.003 | 0.003 | 0.008 | 0.025 | 0.414 | 1.091 | 0.733 |
| *g__Hydrogenoanaerobacterium* | 0.000 | 0.000 | 0.674 | 0.379 | 0.004 | 0.025 | -1.007 | -0.408 | -0.674 |
| *g__Nosocomiicoccus* | 0.610 | 1.109 | 0.000 | 0.000 | 0.005 | 0.025 | 0.086 | 1.528 | 0.610 |
| *g__Ruminococcaceae_UCG-014* | 0.001 | 0.002 | 0.604 | 0.287 | 0.007 | 0.025 | -0.829 | -0.365 | -0.603 |
| *g__Dysgonomonas* | 0.000 | 0.000 | 0.471 | 0.276 | 0.004 | 0.025 | -0.675 | -0.271 | -0.471 |
| *g__Corynebacterium_1* | 0.434 | 0.326 | 0.009 | 0.018 | 0.014 | 0.036 | 0.214 | 0.677 | 0.425 |
| *g__Coprobacillus* | 0.007 | 0.010 | 0.433 | 0.175 | 0.008 | 0.025 | -0.584 | -0.323 | -0.425 |
| *g__Pseudomonas* | 0.303 | 0.282 | 0.061 | 0.022 | 0.014 | 0.036 | 0.087 | 0.477 | 0.241 |
| *g__Stenotrophomonas* | 0.275 | 0.150 | 0.055 | 0.035 | 0.008 | 0.025 | 0.110 | 0.334 | 0.220 |
| *g__Nocardioides* | 0.323 | 0.188 | 0.004 | 0.003 | 0.008 | 0.025 | 0.183 | 0.455 | 0.319 |
| *g__norank_f__FamilyI_o__SubsectionIV* | 0.000 | 0.000 | 0.284 | 0.449 | 0.004 | 0.025 | -0.689 | -0.064 | -0.284 |
| *g__Kurthia* | 0.244 | 0.134 | 0.001 | 0.002 | 0.008 | 0.025 | 0.146 | 0.344 | 0.242 |
| *g__Janibacter* | 0.238 | 0.123 | 0.002 | 0.003 | 0.007 | 0.025 | 0.150 | 0.325 | 0.236 |
| *g__norank_f__Clostridiales_vadinBB60_group* | 0.000 | 0.000 | 0.234 | 0.195 | 0.004 | 0.025 | -0.403 | -0.122 | -0.234 |
| *g__Sporosarcina* | 0.221 | 0.193 | 0.001 | 0.002 | 0.007 | 0.025 | 0.093 | 0.372 | 0.220 |
| *g__Romboutsia* | 0.020 | 0.022 | 0.201 | 0.125 | 0.014 | 0.036 | -0.278 | -0.078 | -0.181 |
| *g__Burkholderia-Paraburkholderia* | 0.204 | 0.384 | 0.000 | 0.000 | 0.017 | 0.036 | 0.013 | 0.526 | 0.204 |
| *g__Chryseobacterium* | 0.192 | 0.321 | 0.000 | 0.000 | 0.005 | 0.025 | 0.026 | 0.463 | 0.192 |
| *g__Paenibacillus* | 0.177 | 0.176 | 0.010 | 0.004 | 0.008 | 0.025 | 0.061 | 0.314 | 0.167 |
| *g__norank_o__JG30-KF-CM45* | 0.161 | 0.076 | 0.015 | 0.009 | 0.008 | 0.025 | 0.100 | 0.212 | 0.146 |
| *g__Caproiciproducens* | 0.012 | 0.019 | 0.161 | 0.062 | 0.007 | 0.025 | -0.196 | -0.091 | -0.149 |
| *g__Ruminococcaceae_UCG-013* | 0.000 | 0.000 | 0.159 | 0.053 | 0.004 | 0.025 | -0.200 | -0.118 | -0.159 |
| *g__Brachybacterium* | 0.153 | 0.117 | 0.000 | 0.000 | 0.005 | 0.025 | 0.079 | 0.251 | 0.153 |
| *g__Streptococcus* | 0.014 | 0.031 | 0.125 | 0.078 | 0.022 | 0.046 | -0.180 | -0.049 | -0.110 |
| *g__norank_f__Coriobacteriaceae* | 0.000 | 0.000 | 0.133 | 0.058 | 0.004 | 0.025 | -0.180 | -0.091 | -0.133 |
| *g__norank_f__Elev-16S-1332* | 0.123 | 0.106 | 0.006 | 0.007 | 0.008 | 0.025 | 0.050 | 0.202 | 0.116 |
| *g__Gaiella* | 0.126 | 0.151 | 0.001 | 0.002 | 0.007 | 0.025 | 0.039 | 0.254 | 0.125 |
| *g__Tyzzerella* | 0.000 | 0.001 | 0.119 | 0.023 | 0.005 | 0.025 | -0.137 | -0.098 | -0.119 |
| *g__norank_p__Saccharibacteria* | 0.110 | 0.066 | 0.008 | 0.008 | 0.008 | 0.025 | 0.052 | 0.151 | 0.102 |
| *g__Coprococcus_1* | 0.000 | 0.000 | 0.111 | 0.037 | 0.004 | 0.025 | -0.139 | -0.080 | -0.111 |
| *g__Gordonibacter* | 0.012 | 0.024 | 0.098 | 0.070 | 0.022 | 0.046 | -0.144 | -0.026 | -0.086 |
| *g__Sphingobium* | 0.110 | 0.213 | 0.000 | 0.000 | 0.017 | 0.036 | 0.009 | 0.289 | 0.110 |
| *g__Candidatus_Soleaferrea* | 0.005 | 0.010 | 0.101 | 0.040 | 0.008 | 0.025 | -0.130 | -0.066 | -0.097 |
| *g__Curtobacterium* | 0.102 | 0.053 | 0.004 | 0.004 | 0.008 | 0.025 | 0.059 | 0.134 | 0.098 |
| *g__Carnobacterium* | 0.000 | 0.001 | 0.105 | 0.035 | 0.005 | 0.025 | -0.131 | -0.079 | -0.105 |
| *g__Kitasatospora* | 0.100 | 0.083 | 0.006 | 0.006 | 0.008 | 0.025 | 0.037 | 0.157 | 0.094 |
| *g__norank_f__Ruminococcaceae* | 0.010 | 0.011 | 0.080 | 0.018 | 0.008 | 0.025 | -0.086 | -0.052 | -0.070 |
| *g__Eubacterium* | 0.002 | 0.002 | 0.085 | 0.038 | 0.008 | 0.025 | -0.118 | -0.060 | -0.083 |
| *g__Eggerthella* | 0.000 | 0.000 | 0.082 | 0.062 | 0.004 | 0.025 | -0.135 | -0.038 | -0.082 |
| *g__Ruminiclostridium_9* | 0.000 | 0.000 | 0.078 | 0.090 | 0.004 | 0.025 | -0.163 | -0.029 | -0.078 |
| *g__Achromobacter* | 0.064 | 0.049 | 0.003 | 0.002 | 0.008 | 0.025 | 0.029 | 0.100 | 0.061 |
| *g__Methylobacterium* | 0.013 | 0.007 | 0.052 | 0.033 | 0.022 | 0.046 | -0.065 | -0.015 | -0.040 |
| *g__Ignatzschineria* | 0.065 | 0.136 | 0.000 | 0.000 | 0.017 | 0.036 | 0.004 | 0.177 | 0.065 |
| *g__unclassified_f__FamilyI_o__SubsectionIV* | 0.000 | 0.000 | 0.063 | 0.084 | 0.004 | 0.025 | -0.139 | -0.019 | -0.063 |
| *g__Kocuria* | 0.063 | 0.137 | 0.000 | 0.000 | 0.005 | 0.025 | 0.004 | 0.175 | 0.063 |
| *g__Flavobacterium* | 0.063 | 0.084 | 0.000 | 0.000 | 0.017 | 0.036 | 0.014 | 0.135 | 0.063 |
| *g__Paracoccus* | 0.056 | 0.032 | 0.006 | 0.005 | 0.014 | 0.036 | 0.027 | 0.074 | 0.050 |
| *g__Exiguobacterium* | 0.062 | 0.045 | 0.000 | 0.000 | 0.005 | 0.025 | 0.031 | 0.094 | 0.062 |
| *g__Streptomyces* | 0.060 | 0.068 | 0.001 | 0.002 | 0.007 | 0.025 | 0.022 | 0.116 | 0.059 |
| *g__Blastococcus* | 0.054 | 0.039 | 0.006 | 0.005 | 0.014 | 0.036 | 0.018 | 0.077 | 0.047 |
| *g__Arthrobacter* | 0.058 | 0.043 | 0.002 | 0.003 | 0.007 | 0.025 | 0.028 | 0.087 | 0.056 |
| *g__norank_o__JG30-KF-AS9* | 0.056 | 0.054 | 0.000 | 0.000 | 0.017 | 0.036 | 0.021 | 0.100 | 0.056 |
| *g__unclassified_p__Firmicutes* | 0.000 | 0.000 | 0.054 | 0.047 | 0.004 | 0.025 | -0.096 | -0.027 | -0.054 |
| *g__Pseudogracilibacillus* | 0.054 | 0.048 | 0.000 | 0.000 | 0.005 | 0.025 | 0.021 | 0.091 | 0.054 |
| *g__Myroides* | 0.051 | 0.040 | 0.000 | 0.000 | 0.005 | 0.025 | 0.026 | 0.081 | 0.051 |
| *g__Lysinibacillus* | 0.048 | 0.055 | 0.003 | 0.005 | 0.013 | 0.036 | 0.014 | 0.094 | 0.045 |
| *g__Shewanella* | 0.047 | 0.048 | 0.000 | 0.000 | 0.017 | 0.036 | 0.015 | 0.085 | 0.047 |
| *g__Brevibacterium* | 0.047 | 0.045 | 0.000 | 0.000 | 0.005 | 0.025 | 0.018 | 0.084 | 0.047 |
| *g__Tissierella* | 0.042 | 0.036 | 0.000 | 0.000 | 0.005 | 0.025 | 0.017 | 0.067 | 0.042 |
| *g__Marmoricola* | 0.040 | 0.032 | 0.000 | 0.000 | 0.017 | 0.036 | 0.017 | 0.061 | 0.040 |
| *g__Proteus* | 0.039 | 0.038 | 0.000 | 0.000 | 0.005 | 0.025 | 0.013 | 0.068 | 0.039 |
| *g__Sphingomonas* | 0.037 | 0.075 | 0.001 | 0.001 | 0.020 | 0.042 | 0.003 | 0.099 | 0.037 |
| *g__Legionella* | 0.035 | 0.021 | 0.000 | 0.000 | 0.005 | 0.025 | 0.021 | 0.050 | 0.035 |
| *g__Williamsia* | 0.033 | 0.024 | 0.001 | 0.002 | 0.007 | 0.025 | 0.015 | 0.049 | 0.033 |
| *g__norank_o__Gaiellales* | 0.032 | 0.023 | 0.002 | 0.004 | 0.007 | 0.025 | 0.016 | 0.050 | 0.031 |
| *g__unclassified_f__Bacillaceae* | 0.032 | 0.024 | 0.000 | 0.000 | 0.005 | 0.025 | 0.017 | 0.051 | 0.032 |
| *g__norank_o__Acidimicrobiales* | 0.029 | 0.023 | 0.002 | 0.003 | 0.007 | 0.025 | 0.012 | 0.046 | 0.028 |
| *g__Leuconostoc* | 0.002 | 0.005 | 0.028 | 0.021 | 0.007 | 0.025 | -0.044 | -0.012 | -0.026 |
| *g__unclassified_f__Geodermatophilaceae* | 0.030 | 0.030 | 0.000 | 0.000 | 0.005 | 0.025 | 0.013 | 0.056 | 0.030 |
| *g__Neomicrococcus* | 0.030 | 0.033 | 0.000 | 0.000 | 0.005 | 0.025 | 0.009 | 0.054 | 0.030 |
| *g__norank_f__DA111* | 0.029 | 0.033 | 0.000 | 0.000 | 0.005 | 0.025 | 0.008 | 0.055 | 0.029 |
| *g__Microcoleus* | 0.028 | 0.054 | 0.000 | 0.000 | 0.017 | 0.036 | 0.004 | 0.072 | 0.028 |
| *g__Intestinimonas* | 0.000 | 0.000 | 0.027 | 0.020 | 0.004 | 0.025 | -0.042 | -0.012 | -0.027 |
| *g__unclassified_f__Dermacoccaceae* | 0.026 | 0.013 | 0.000 | 0.000 | 0.005 | 0.025 | 0.016 | 0.035 | 0.026 |
| *g__Geobacillus* | 0.001 | 0.003 | 0.025 | 0.021 | 0.010 | 0.030 | -0.040 | -0.008 | -0.024 |
| *g__Aeromicrobium* | 0.025 | 0.012 | 0.001 | 0.002 | 0.007 | 0.025 | 0.016 | 0.033 | 0.024 |
| *g__norank_f__Limnochordaceae* | 0.025 | 0.012 | 0.000 | 0.000 | 0.005 | 0.025 | 0.017 | 0.035 | 0.025 |
| *g__unclassified_f__Parachlamydiaceae* | 0.024 | 0.017 | 0.001 | 0.002 | 0.007 | 0.025 | 0.011 | 0.038 | 0.023 |
| *g__Anaerofustis* | 0.000 | 0.000 | 0.025 | 0.013 | 0.004 | 0.025 | -0.033 | -0.013 | -0.025 |
| *g__Geodermatophilus* | 0.024 | 0.023 | 0.000 | 0.000 | 0.005 | 0.025 | 0.010 | 0.044 | 0.024 |
| *g__norank_f__Peptococcaceae* | 0.000 | 0.000 | 0.023 | 0.011 | 0.004 | 0.025 | -0.031 | -0.015 | -0.023 |
| *g__norank_f__Bacillaceae* | 0.022 | 0.015 | 0.000 | 0.000 | 0.005 | 0.025 | 0.013 | 0.033 | 0.022 |
| *g__Catabacter* | 0.000 | 0.001 | 0.022 | 0.017 | 0.005 | 0.025 | -0.037 | -0.012 | -0.022 |
| *g__Planifilum* | 0.021 | 0.020 | 0.000 | 0.000 | 0.005 | 0.025 | 0.008 | 0.036 | 0.021 |
| *g__unclassified_o__Clostridiales* | 0.000 | 0.000 | 0.021 | 0.011 | 0.004 | 0.025 | -0.031 | -0.013 | -0.021 |
| *g__Ureaplasma* | 0.021 | 0.021 | 0.000 | 0.000 | 0.005 | 0.025 | 0.007 | 0.037 | 0.021 |
| *g__Mobilitalea* | 0.019 | 0.016 | 0.001 | 0.002 | 0.021 | 0.045 | 0.006 | 0.029 | 0.018 |
| *g__Rummeliibacillus* | 0.020 | 0.013 | 0.000 | 0.000 | 0.005 | 0.025 | 0.010 | 0.029 | 0.020 |
| *g__Oscillibacter* | 0.000 | 0.000 | 0.019 | 0.016 | 0.004 | 0.025 | -0.032 | -0.009 | -0.019 |
| *g__Solirubrobacter* | 0.019 | 0.018 | 0.000 | 0.000 | 0.005 | 0.025 | 0.007 | 0.033 | 0.019 |
| *g__unclassified_f__Family_XIII* | 0.000 | 0.000 | 0.019 | 0.016 | 0.004 | 0.025 | -0.033 | -0.009 | -0.019 |
| *g__Macrococcus* | 0.019 | 0.017 | 0.000 | 0.000 | 0.005 | 0.025 | 0.008 | 0.032 | 0.019 |
| *g__Butyricicoccus* | 0.000 | 0.000 | 0.019 | 0.017 | 0.015 | 0.036 | -0.032 | -0.006 | -0.019 |
| *g__norank_f__Thermoactinomycetaceae* | 0.018 | 0.015 | 0.000 | 0.000 | 0.005 | 0.025 | 0.008 | 0.029 | 0.018 |
| *g__unclassified_f__Streptosporangiaceae* | 0.017 | 0.010 | 0.000 | 0.000 | 0.005 | 0.025 | 0.011 | 0.025 | 0.017 |
| *g__Oxalobacter* | 0.000 | 0.000 | 0.017 | 0.016 | 0.015 | 0.036 | -0.030 | -0.005 | -0.017 |
| *g__Nostoc* | 0.000 | 0.000 | 0.015 | 0.014 | 0.015 | 0.036 | -0.027 | -0.006 | -0.015 |
| *g__norank_o__HTA4* | 0.003 | 0.004 | 0.012 | 0.005 | 0.022 | 0.046 | -0.013 | -0.003 | -0.008 |
| *g__unclassified_f__Christensenellaceae* | 0.000 | 0.000 | 0.015 | 0.011 | 0.015 | 0.036 | -0.023 | -0.006 | -0.015 |
| *g__Anaerofilum* | 0.000 | 0.000 | 0.014 | 0.011 | 0.004 | 0.025 | -0.023 | -0.005 | -0.014 |
| *g__Mycoplasma* | 0.014 | 0.011 | 0.000 | 0.000 | 0.005 | 0.025 | 0.006 | 0.022 | 0.014 |
| *g__Solibacillus* | 0.014 | 0.022 | 0.000 | 0.000 | 0.017 | 0.036 | 0.003 | 0.032 | 0.014 |
| *g__norank_f__Christensenellaceae* | 0.000 | 0.000 | 0.013 | 0.010 | 0.004 | 0.025 | -0.021 | -0.005 | -0.013 |
| *g__Turicibacter* | 0.013 | 0.011 | 0.000 | 0.000 | 0.005 | 0.025 | 0.006 | 0.022 | 0.013 |
| *g__norank_f__Xanthomonadaceae* | 0.012 | 0.009 | 0.001 | 0.001 | 0.012 | 0.035 | 0.006 | 0.019 | 0.011 |
| *g__Jeotgalicoccus* | 0.012 | 0.015 | 0.000 | 0.000 | 0.017 | 0.036 | 0.003 | 0.025 | 0.012 |
| *g__Anaerosalibacter* | 0.012 | 0.010 | 0.000 | 0.000 | 0.005 | 0.025 | 0.005 | 0.020 | 0.012 |
| *g__Ammoniphilus* | 0.012 | 0.012 | 0.000 | 0.000 | 0.005 | 0.025 | 0.005 | 0.022 | 0.012 |
| *g__Clostridium_sensu_stricto_13* | 0.010 | 0.005 | 0.002 | 0.002 | 0.008 | 0.025 | 0.004 | 0.012 | 0.008 |
| *g__Ruminiclostridium* | 0.000 | 0.000 | 0.012 | 0.008 | 0.004 | 0.025 | -0.018 | -0.006 | -0.012 |
| *g__Atopostipes* | 0.012 | 0.017 | 0.000 | 0.000 | 0.017 | 0.036 | 0.002 | 0.026 | 0.012 |
| *g__unclassified_f__Intrasporangiaceae* | 0.011 | 0.011 | 0.000 | 0.000 | 0.005 | 0.025 | 0.004 | 0.020 | 0.011 |
| *g__Actinomadura* | 0.011 | 0.017 | 0.000 | 0.000 | 0.017 | 0.036 | 0.003 | 0.026 | 0.011 |
| *g__norank_f__p35j06ok* | 0.011 | 0.019 | 0.000 | 0.000 | 0.017 | 0.036 | 0.001 | 0.027 | 0.011 |
| *g__unclassified_f__Paenibacillaceae* | 0.011 | 0.009 | 0.000 | 0.000 | 0.005 | 0.025 | 0.004 | 0.018 | 0.011 |
| *g__unclassified_f__Hyphomicrobiaceae* | 0.009 | 0.009 | 0.001 | 0.002 | 0.012 | 0.035 | 0.003 | 0.016 | 0.008 |
| *g__Flexivirga* | 0.010 | 0.006 | 0.000 | 0.000 | 0.017 | 0.036 | 0.006 | 0.015 | 0.010 |
| *g__Bhargavaea* | 0.010 | 0.009 | 0.000 | 0.000 | 0.017 | 0.036 | 0.004 | 0.016 | 0.010 |
| *g__Delftia* | 0.009 | 0.010 | 0.000 | 0.000 | 0.005 | 0.025 | 0.003 | 0.016 | 0.009 |
| *g__unclassified_o__Micrococcales* | 0.009 | 0.007 | 0.000 | 0.000 | 0.017 | 0.036 | 0.004 | 0.014 | 0.009 |
| *g__Tuberibacillus* | 0.009 | 0.005 | 0.000 | 0.000 | 0.005 | 0.025 | 0.005 | 0.013 | 0.009 |
| *g__unclassified_o__Chlamydiales* | 0.008 | 0.007 | 0.001 | 0.002 | 0.012 | 0.035 | 0.003 | 0.013 | 0.007 |
| *g__norank_c__KD4-96* | 0.009 | 0.013 | 0.000 | 0.000 | 0.017 | 0.036 | 0.002 | 0.020 | 0.009 |
| *g__Symbiobacterium* | 0.008 | 0.008 | 0.000 | 0.000 | 0.017 | 0.036 | 0.003 | 0.014 | 0.008 |
| *g__Ruminococcaceae_NK4A214_group* | 0.000 | 0.000 | 0.008 | 0.005 | 0.004 | 0.025 | -0.012 | -0.005 | -0.008 |
| *g__unclassified_f__Acetobacteraceae* | 0.008 | 0.009 | 0.000 | 0.000 | 0.017 | 0.036 | 0.002 | 0.016 | 0.008 |
| *g__Anabaena* | 0.000 | 0.000 | 0.008 | 0.007 | 0.015 | 0.036 | -0.014 | -0.003 | -0.008 |
| *g__Thermobacillus* | 0.007 | 0.008 | 0.000 | 0.000 | 0.017 | 0.036 | 0.002 | 0.013 | 0.007 |
| *g__Agaricicola* | 0.007 | 0.005 | 0.000 | 0.000 | 0.017 | 0.036 | 0.003 | 0.011 | 0.007 |
| *g__Enteractinococcus* | 0.007 | 0.006 | 0.000 | 0.000 | 0.017 | 0.036 | 0.002 | 0.011 | 0.007 |
| *g__Tepidimicrobium* | 0.006 | 0.004 | 0.000 | 0.000 | 0.017 | 0.036 | 0.004 | 0.009 | 0.006 |
| *g__norank_c__Bacilli* | 0.006 | 0.004 | 0.000 | 0.000 | 0.005 | 0.025 | 0.004 | 0.009 | 0.006 |
| *g__Nitrolancea* | 0.006 | 0.006 | 0.000 | 0.000 | 0.017 | 0.036 | 0.002 | 0.011 | 0.006 |
| *g__Sphaerisporangium* | 0.006 | 0.004 | 0.000 | 0.000 | 0.017 | 0.036 | 0.003 | 0.009 | 0.006 |
| *g__norank_o__MBA03* | 0.006 | 0.006 | 0.000 | 0.000 | 0.005 | 0.025 | 0.002 | 0.010 | 0.006 |
| *g__Ruminococcaceae_UCG-005* | 0.000 | 0.000 | 0.006 | 0.005 | 0.015 | 0.036 | -0.010 | -0.002 | -0.006 |
| *g__Streptosporangium* | 0.005 | 0.007 | 0.000 | 0.000 | 0.017 | 0.036 | 0.001 | 0.011 | 0.005 |
| *g__[Eubacterium]_nodatum_group* | 0.000 | 0.000 | 0.005 | 0.003 | 0.015 | 0.036 | -0.007 | -0.002 | -0.005 |
| *g__Filobacterium* | 0.005 | 0.003 | 0.000 | 0.000 | 0.005 | 0.025 | 0.002 | 0.007 | 0.005 |
| *g__norank_o__C178B* | 0.004 | 0.004 | 0.000 | 0.000 | 0.017 | 0.036 | 0.002 | 0.007 | 0.004 |
| *g__Salana* | 0.004 | 0.003 | 0.000 | 0.000 | 0.017 | 0.036 | 0.002 | 0.007 | 0.004 |
| *g__Actinoallomurus* | 0.004 | 0.005 | 0.000 | 0.000 | 0.017 | 0.036 | 0.001 | 0.008 | 0.004 |
| *g__Ornithinimicrobium* | 0.004 | 0.003 | 0.000 | 0.000 | 0.017 | 0.036 | 0.001 | 0.006 | 0.004 |

**Table S2** Differences between the DS and HB groups at the genus level (Wilcoxon rank-sum test, *P* < 0.05).

| Species name | DS-Mean (%) | DS-Sd (%) | HB-Mean (%) | HB-Sd (%) | *P* value | Corrected *p* value | Lower ci | Upper ci | Effectsize |
| --- | --- | --- | --- | --- | --- | --- | --- | --- | --- |
| *g__Bacteroides* | 3.961 | 8.948 | 33.950 | 22.340 | 0.008 | 0.019 | -45.940 | -12.330 | -29.980 |
| *g__Deefgea* | 0.000 | 0.000 | 16.520 | 19.040 | 0.002 | 0.010 | -31.980 | -3.470 | -16.520 |
| *g__Citrobacter* | 13.300 | 3.482 | 0.147 | 0.180 | 0.003 | 0.012 | 10.540 | 15.700 | 13.150 |
| *g__Enterococcus* | 11.450 | 8.619 | 0.002 | 0.003 | 0.003 | 0.010 | 6.307 | 18.800 | 11.450 |
| *g__Vagococcus* | 8.616 | 7.891 | 0.000 | 0.000 | 0.001 | 0.010 | 4.029 | 14.580 | 8.616 |
| *g__Hafnia-Obesumbacterium* | 4.507 | 3.340 | 1.130 | 1.832 | 0.027 | 0.048 | 0.488 | 6.191 | 3.377 |
| *g__unclassified_f__Micrococcaceae* | 4.724 | 2.539 | 0.015 | 0.028 | 0.003 | 0.012 | 2.906 | 6.523 | 4.709 |
| *g__Providencia* | 3.965 | 7.067 | 0.000 | 0.001 | 0.002 | 0.010 | 0.922 | 9.767 | 3.965 |
| *g__Morganella* | 3.712 | 1.334 | 0.002 | 0.005 | 0.002 | 0.010 | 2.740 | 4.798 | 3.710 |
| *g__Staphylococcus* | 3.621 | 4.338 | 0.002 | 0.002 | 0.003 | 0.012 | 1.197 | 7.073 | 3.619 |
| *g__[Eubacterium]_fissicatena_group* | 0.003 | 0.004 | 3.145 | 4.829 | 0.003 | 0.012 | -7.060 | -0.697 | -3.142 |
| *g__Aeromonas* | 2.971 | 1.599 | 0.058 | 0.097 | 0.003 | 0.012 | 1.741 | 4.012 | 2.913 |
| *g__Lactococcus* | 2.762 | 2.462 | 0.008 | 0.014 | 0.003 | 0.010 | 1.166 | 4.842 | 2.755 |
| *g__Rhodococcus* | 2.066 | 1.037 | 0.074 | 0.117 | 0.003 | 0.012 | 1.250 | 2.711 | 1.992 |
| *g__Robinsoniella* | 0.001 | 0.002 | 2.096 | 2.098 | 0.003 | 0.012 | -3.533 | -0.705 | -2.095 |
| *g__Acinetobacter* | 1.896 | 3.433 | 0.031 | 0.064 | 0.007 | 0.017 | 0.178 | 4.701 | 1.865 |
| *g__Odoribacter* | 0.000 | 0.000 | 1.712 | 2.148 | 0.002 | 0.010 | -3.382 | -0.522 | -1.712 |
| *g__Plesiomonas* | 1.616 | 1.609 | 0.000 | 0.001 | 0.002 | 0.010 | 0.649 | 2.933 | 1.616 |
| *g__Koukoulia* | 1.550 | 1.120 | 0.000 | 0.000 | 0.001 | 0.010 | 0.834 | 2.511 | 1.550 |
| *g__Tyzzerella* | 0.000 | 0.001 | 1.545 | 1.870 | 0.003 | 0.010 | -3.013 | -0.327 | -1.545 |
| *g__Alistipes* | 0.001 | 0.001 | 1.505 | 1.284 | 0.003 | 0.012 | -2.410 | -0.631 | -1.505 |
| *g__Eubacterium* | 0.002 | 0.002 | 1.486 | 1.404 | 0.003 | 0.012 | -2.540 | -0.595 | -1.483 |
| *g__Lactobacillus* | 1.479 | 3.411 | 0.000 | 0.000 | 0.001 | 0.010 | 0.033 | 4.273 | 1.479 |
| *g__unclassified_f__Oxalobacteraceae* | 1.475 | 2.750 | 0.000 | 0.000 | 0.001 | 0.010 | 0.113 | 3.792 | 1.475 |
| *g__Leucobacter* | 1.455 | 0.905 | 0.002 | 0.005 | 0.002 | 0.010 | 0.865 | 2.205 | 1.452 |
| *g__Comamonas* | 1.395 | 1.445 | 0.010 | 0.022 | 0.005 | 0.014 | 0.206 | 2.580 | 1.385 |
| *g__Lachnoclostridium* | 0.004 | 0.005 | 1.371 | 1.440 | 0.003 | 0.012 | -2.473 | -0.410 | -1.367 |
| *g__norank_c__Cyanobacteria* | 0.986 | 0.483 | 0.236 | 0.615 | 0.026 | 0.047 | 0.186 | 1.266 | 0.749 |
| *g__Ruminococcaceae_UCG-009* | 0.009 | 0.021 | 1.122 | 1.404 | 0.004 | 0.014 | -2.171 | -0.276 | -1.113 |
| *g__Pseudochrobactrum* | 1.065 | 0.866 | 0.000 | 0.001 | 0.002 | 0.010 | 0.493 | 1.750 | 1.065 |
| *g__Anaerosinus* | 0.000 | 0.000 | 1.045 | 2.007 | 0.018 | 0.034 | -2.612 | -0.005 | -1.045 |
| *g__Escherichia-Shigella* | 0.977 | 0.554 | 0.001 | 0.002 | 0.003 | 0.010 | 0.625 | 1.416 | 0.976 |
| *g__Microbacterium* | 0.950 | 0.477 | 0.011 | 0.028 | 0.003 | 0.010 | 0.567 | 1.265 | 0.939 |
| *g__Oerskovia* | 0.940 | 0.791 | 0.000 | 0.001 | 0.002 | 0.010 | 0.337 | 1.550 | 0.940 |
| *g__unclassified_f__Flavobacteriaceae* | 0.000 | 0.000 | 0.817 | 2.130 | 0.018 | 0.034 | -2.428 | -0.003 | -0.817 |
| *g__unclassified_f__Enterobacteriaceae* | 0.780 | 0.890 | 0.001 | 0.002 | 0.003 | 0.010 | 0.207 | 1.501 | 0.779 |
| *g__Glutamicibacter* | 0.736 | 0.472 | 0.000 | 0.000 | 0.001 | 0.010 | 0.425 | 1.083 | 0.736 |
| *g__Rikenella* | 0.000 | 0.000 | 0.724 | 1.062 | 0.002 | 0.010 | -1.524 | -0.130 | -0.724 |
| *g__Gordonibacter* | 0.012 | 0.024 | 0.677 | 1.143 | 0.003 | 0.012 | -1.559 | -0.139 | -0.665 |
| *g__Peptoclostridium* | 0.646 | 1.118 | 0.000 | 0.000 | 0.001 | 0.010 | 0.103 | 1.578 | 0.646 |
| *g__Nosocomiicoccus* | 0.610 | 1.109 | 0.000 | 0.000 | 0.001 | 0.010 | 0.091 | 1.530 | 0.610 |
| *g__Bilophila* | 0.000 | 0.000 | 0.533 | 1.000 | 0.002 | 0.010 | -1.322 | -0.078 | -0.533 |
| *g__Hydrogenoanaerobacterium* | 0.000 | 0.000 | 0.508 | 0.861 | 0.002 | 0.010 | -1.218 | -0.069 | -0.508 |
| *g__Anaerofilum* | 0.000 | 0.000 | 0.488 | 0.949 | 0.018 | 0.034 | -1.194 | -0.030 | -0.488 |
| *g__Corynebacterium_1* | 0.434 | 0.326 | 0.000 | 0.001 | 0.002 | 0.010 | 0.217 | 0.692 | 0.434 |
| *g__Bacillus* | 0.418 | 0.267 | 0.007 | 0.014 | 0.003 | 0.012 | 0.212 | 0.607 | 0.412 |
| *g__Parabacteroides* | 0.000 | 0.001 | 0.365 | 0.405 | 0.003 | 0.010 | -0.644 | -0.112 | -0.365 |
| *g__norank_f__Family_XIII* | 0.000 | 0.001 | 0.350 | 0.414 | 0.003 | 0.010 | -0.650 | -0.025 | -0.349 |
| *g__Desulfovibrio* | 0.011 | 0.016 | 0.335 | 0.501 | 0.018 | 0.034 | -0.723 | -0.079 | -0.325 |
| *g__Nocardioides* | 0.323 | 0.188 | 0.001 | 0.002 | 0.002 | 0.010 | 0.193 | 0.460 | 0.322 |
| *g__Stenotrophomonas* | 0.275 | 0.150 | 0.002 | 0.003 | 0.003 | 0.010 | 0.171 | 0.390 | 0.273 |
| *g__Kurthia* | 0.244 | 0.134 | 0.000 | 0.000 | 0.001 | 0.010 | 0.151 | 0.345 | 0.244 |
| *g__Janibacter* | 0.238 | 0.123 | 0.003 | 0.008 | 0.002 | 0.010 | 0.150 | 0.326 | 0.235 |
| *g__Fusobacterium* | 0.230 | 0.498 | 0.000 | 0.000 | 0.006 | 0.014 | 0.010 | 0.638 | 0.230 |
| *g__Sporosarcina* | 0.221 | 0.193 | 0.000 | 0.001 | 0.002 | 0.010 | 0.102 | 0.382 | 0.221 |
| *g__Empedobacter* | 0.219 | 0.198 | 0.000 | 0.000 | 0.001 | 0.010 | 0.083 | 0.372 | 0.219 |
| *g__Acidovorax* | 0.202 | 0.376 | 0.000 | 0.001 | 0.002 | 0.010 | 0.013 | 0.511 | 0.202 |
| *g__Akkermansia* | 0.000 | 0.000 | 0.186 | 0.257 | 0.007 | 0.016 | -0.372 | -0.032 | -0.186 |
| *g__Paenibacillus* | 0.177 | 0.176 | 0.001 | 0.002 | 0.003 | 0.010 | 0.068 | 0.325 | 0.175 |
| *g__Ruminiclostridium_5* | 0.019 | 0.047 | 0.154 | 0.175 | 0.017 | 0.034 | -0.286 | -0.022 | -0.135 |
| *g__Rhizobium* | 0.165 | 0.193 | 0.004 | 0.008 | 0.005 | 0.014 | 0.058 | 0.324 | 0.161 |
| *g__Butyricimonas* | 0.000 | 0.000 | 0.169 | 0.254 | 0.002 | 0.010 | -0.367 | -0.039 | -0.169 |
| *g__norank_o__JG30-KF-CM45* | 0.161 | 0.076 | 0.004 | 0.010 | 0.002 | 0.010 | 0.108 | 0.219 | 0.157 |
| *g__Brachybacterium* | 0.153 | 0.117 | 0.000 | 0.000 | 0.001 | 0.010 | 0.075 | 0.246 | 0.153 |
| *g__Gaiella* | 0.126 | 0.151 | 0.001 | 0.002 | 0.002 | 0.010 | 0.040 | 0.251 | 0.125 |
| *g__norank_f__Elev-16S-1332* | 0.123 | 0.106 | 0.003 | 0.006 | 0.003 | 0.010 | 0.055 | 0.210 | 0.120 |
| *g__Mycobacterium* | 0.108 | 0.073 | 0.018 | 0.036 | 0.012 | 0.027 | 0.032 | 0.152 | 0.090 |
| *g__Sphingobium* | 0.110 | 0.213 | 0.000 | 0.001 | 0.010 | 0.023 | 0.009 | 0.285 | 0.110 |
| *g__norank_p__Saccharibacteria* | 0.110 | 0.066 | 0.000 | 0.000 | 0.001 | 0.010 | 0.062 | 0.155 | 0.110 |
| *g__Curtobacterium* | 0.102 | 0.053 | 0.007 | 0.017 | 0.004 | 0.014 | 0.056 | 0.138 | 0.095 |
| *g__Kitasatospora* | 0.100 | 0.083 | 0.001 | 0.001 | 0.003 | 0.010 | 0.040 | 0.172 | 0.099 |
| *g__Weissella* | 0.097 | 0.039 | 0.001 | 0.002 | 0.003 | 0.010 | 0.071 | 0.127 | 0.096 |
| *g__Ochrobactrum* | 0.093 | 0.055 | 0.000 | 0.000 | 0.001 | 0.010 | 0.051 | 0.130 | 0.093 |
| *g__Ruminococcaceae_NK4A214_group* | 0.000 | 0.000 | 0.085 | 0.096 | 0.018 | 0.034 | -0.158 | -0.024 | -0.085 |
| *g__Bosea* | 0.075 | 0.046 | 0.000 | 0.001 | 0.002 | 0.010 | 0.040 | 0.108 | 0.075 |
| *g__Sphingobacterium* | 0.073 | 0.105 | 0.000 | 0.000 | 0.001 | 0.010 | 0.016 | 0.160 | 0.073 |
| *g__Paracoccus* | 0.056 | 0.032 | 0.011 | 0.027 | 0.025 | 0.045 | 0.015 | 0.073 | 0.045 |
| *g__Achromobacter* | 0.064 | 0.049 | 0.002 | 0.003 | 0.003 | 0.010 | 0.030 | 0.102 | 0.062 |
| *g__Ignatzschineria* | 0.065 | 0.136 | 0.000 | 0.000 | 0.006 | 0.014 | 0.004 | 0.178 | 0.065 |
| *g__Kocuria* | 0.063 | 0.137 | 0.001 | 0.003 | 0.009 | 0.020 | 0.002 | 0.174 | 0.061 |
| *g__Exiguobacterium* | 0.062 | 0.045 | 0.000 | 0.000 | 0.001 | 0.010 | 0.030 | 0.093 | 0.062 |
| *g__Oscillibacter* | 0.000 | 0.000 | 0.061 | 0.080 | 0.007 | 0.016 | -0.120 | -0.013 | -0.061 |
| *g__Streptomyces* | 0.060 | 0.068 | 0.000 | 0.000 | 0.001 | 0.010 | 0.023 | 0.120 | 0.060 |
| *g__norank_o__JG30-KF-AS9* | 0.056 | 0.054 | 0.000 | 0.000 | 0.006 | 0.014 | 0.020 | 0.097 | 0.056 |
| *g__Brevundimonas* | 0.053 | 0.091 | 0.002 | 0.002 | 0.023 | 0.041 | 0.006 | 0.130 | 0.052 |
| *g__Blastococcus* | 0.054 | 0.039 | 0.001 | 0.002 | 0.002 | 0.010 | 0.027 | 0.082 | 0.053 |
| *g__Mucispirillum* | 0.000 | 0.000 | 0.054 | 0.059 | 0.002 | 0.010 | -0.098 | -0.018 | -0.054 |
| *g__Anaerovorax* | 0.000 | 0.000 | 0.054 | 0.071 | 0.018 | 0.034 | -0.110 | -0.013 | -0.054 |
| *g__Pseudogracilibacillus* | 0.054 | 0.048 | 0.000 | 0.000 | 0.001 | 0.010 | 0.021 | 0.090 | 0.054 |
| *g__Myroides* | 0.051 | 0.040 | 0.000 | 0.000 | 0.001 | 0.010 | 0.026 | 0.085 | 0.051 |
| *g__Agromyces* | 0.051 | 0.034 | 0.000 | 0.000 | 0.001 | 0.010 | 0.027 | 0.075 | 0.051 |
| *g__[Eubacterium]_nodatum_group* | 0.000 | 0.000 | 0.048 | 0.072 | 0.007 | 0.016 | -0.098 | -0.006 | -0.048 |
| *g__Shewanella* | 0.047 | 0.048 | 0.001 | 0.002 | 0.016 | 0.034 | 0.012 | 0.082 | 0.046 |
| *g__Lysinibacillus* | 0.048 | 0.055 | 0.000 | 0.000 | 0.001 | 0.010 | 0.018 | 0.094 | 0.048 |
| *g__Brevibacterium* | 0.047 | 0.045 | 0.000 | 0.001 | 0.002 | 0.010 | 0.018 | 0.075 | 0.046 |
| *g__Bradyrhizobium* | 0.041 | 0.027 | 0.005 | 0.011 | 0.011 | 0.025 | 0.017 | 0.058 | 0.036 |
| *g__unclassified_f__Micromonosporaceae* | 0.044 | 0.047 | 0.000 | 0.000 | 0.001 | 0.010 | 0.019 | 0.084 | 0.044 |
| *g__unclassified_o__Lactobacillales* | 0.042 | 0.055 | 0.000 | 0.000 | 0.006 | 0.014 | 0.006 | 0.083 | 0.042 |
| *g__Tissierella* | 0.042 | 0.036 | 0.000 | 0.000 | 0.001 | 0.010 | 0.016 | 0.071 | 0.042 |
| *g__Marmoricola* | 0.040 | 0.032 | 0.000 | 0.001 | 0.010 | 0.023 | 0.015 | 0.064 | 0.039 |
| *g__Proteus* | 0.039 | 0.038 | 0.000 | 0.000 | 0.001 | 0.010 | 0.015 | 0.072 | 0.039 |
| *g__Sphingomonas* | 0.037 | 0.075 | 0.000 | 0.001 | 0.005 | 0.014 | 0.003 | 0.099 | 0.037 |
| *g__norank_o__Gaiellales* | 0.032 | 0.023 | 0.005 | 0.012 | 0.015 | 0.034 | 0.011 | 0.047 | 0.027 |
| *g__Legionella* | 0.035 | 0.021 | 0.000 | 0.000 | 0.001 | 0.010 | 0.020 | 0.049 | 0.035 |
| *g__norank_f__Rhodobiaceae* | 0.031 | 0.026 | 0.004 | 0.006 | 0.007 | 0.017 | 0.011 | 0.050 | 0.028 |
| *g__Williamsia* | 0.033 | 0.024 | 0.002 | 0.004 | 0.005 | 0.014 | 0.013 | 0.050 | 0.032 |
| *g__Micromonospora* | 0.033 | 0.025 | 0.000 | 0.001 | 0.002 | 0.010 | 0.014 | 0.052 | 0.033 |
| *g__unclassified_f__Bacillaceae* | 0.032 | 0.024 | 0.000 | 0.000 | 0.001 | 0.010 | 0.017 | 0.051 | 0.032 |
| *g__Gordonia* | 0.031 | 0.028 | 0.000 | 0.000 | 0.001 | 0.010 | 0.013 | 0.055 | 0.031 |
| *g__unclassified_f__Geodermatophilaceae* | 0.030 | 0.030 | 0.000 | 0.000 | 0.001 | 0.010 | 0.012 | 0.054 | 0.030 |
| *g__Neomicrococcus* | 0.030 | 0.033 | 0.000 | 0.000 | 0.001 | 0.010 | 0.009 | 0.057 | 0.030 |
| *g__norank_o__Acidimicrobiales* | 0.029 | 0.023 | 0.000 | 0.000 | 0.001 | 0.010 | 0.014 | 0.047 | 0.029 |
| *g__norank_f__DA111* | 0.029 | 0.033 | 0.000 | 0.000 | 0.001 | 0.010 | 0.007 | 0.057 | 0.029 |
| *g__Microcoleus* | 0.028 | 0.054 | 0.000 | 0.000 | 0.006 | 0.014 | 0.004 | 0.074 | 0.028 |
| *g__Cellulosimicrobium* | 0.028 | 0.029 | 0.000 | 0.000 | 0.019 | 0.034 | 0.007 | 0.051 | 0.028 |
| *g__Acidothermus* | 0.026 | 0.023 | 0.001 | 0.002 | 0.005 | 0.014 | 0.010 | 0.044 | 0.025 |
| *g__unclassified_f__Dermacoccaceae* | 0.026 | 0.013 | 0.000 | 0.000 | 0.001 | 0.010 | 0.017 | 0.035 | 0.026 |
| *g__norank_f__Limnochordaceae* | 0.025 | 0.012 | 0.000 | 0.000 | 0.001 | 0.010 | 0.017 | 0.035 | 0.025 |
| *g__Aeromicrobium* | 0.025 | 0.012 | 0.000 | 0.001 | 0.002 | 0.010 | 0.015 | 0.033 | 0.024 |
| *g__Hydrogenispora* | 0.024 | 0.017 | 0.000 | 0.000 | 0.006 | 0.014 | 0.012 | 0.036 | 0.024 |
| *g__Geodermatophilus* | 0.024 | 0.023 | 0.000 | 0.000 | 0.001 | 0.010 | 0.010 | 0.042 | 0.024 |
| *g__unclassified_f__Parachlamydiaceae* | 0.024 | 0.017 | 0.000 | 0.000 | 0.001 | 0.010 | 0.012 | 0.038 | 0.024 |
| *g__Tumebacillus* | 0.023 | 0.018 | 0.000 | 0.000 | 0.006 | 0.014 | 0.010 | 0.037 | 0.023 |
| *g__norank_f__Bacillaceae* | 0.022 | 0.015 | 0.000 | 0.000 | 0.001 | 0.010 | 0.012 | 0.032 | 0.022 |
| *g__norank_f__Coriobacteriaceae* | 0.000 | 0.000 | 0.022 | 0.027 | 0.007 | 0.016 | -0.042 | -0.006 | -0.022 |
| *g__Planifilum* | 0.021 | 0.020 | 0.000 | 0.000 | 0.001 | 0.010 | 0.008 | 0.037 | 0.021 |
| *g__unclassified_f__Xanthobacteraceae* | 0.018 | 0.015 | 0.003 | 0.008 | 0.013 | 0.029 | 0.002 | 0.028 | 0.015 |
| *g__Ureaplasma* | 0.021 | 0.021 | 0.000 | 0.000 | 0.001 | 0.010 | 0.007 | 0.036 | 0.021 |
| *g__Ensifer* | 0.020 | 0.012 | 0.000 | 0.001 | 0.002 | 0.010 | 0.012 | 0.030 | 0.020 |
| *g__Rummeliibacillus* | 0.020 | 0.013 | 0.000 | 0.000 | 0.001 | 0.010 | 0.010 | 0.030 | 0.020 |
| *g__Mobilitalea* | 0.019 | 0.016 | 0.000 | 0.000 | 0.001 | 0.010 | 0.008 | 0.031 | 0.019 |
| *g__Solirubrobacter* | 0.019 | 0.018 | 0.000 | 0.000 | 0.001 | 0.010 | 0.007 | 0.035 | 0.019 |
| *g__Macrococcus* | 0.019 | 0.017 | 0.000 | 0.000 | 0.001 | 0.010 | 0.007 | 0.032 | 0.019 |
| *g__Alcaligenes* | 0.019 | 0.037 | 0.000 | 0.000 | 0.019 | 0.034 | 0.001 | 0.050 | 0.019 |
| *g__Christensenella* | 0.000 | 0.000 | 0.018 | 0.021 | 0.018 | 0.034 | -0.033 | -0.006 | -0.018 |
| *g__Kaistia* | 0.018 | 0.015 | 0.000 | 0.000 | 0.001 | 0.010 | 0.008 | 0.030 | 0.018 |
| *g__norank_f__Thermoactinomycetaceae* | 0.018 | 0.015 | 0.000 | 0.000 | 0.001 | 0.010 | 0.008 | 0.028 | 0.018 |
| *g__Microlunatus* | 0.017 | 0.019 | 0.001 | 0.002 | 0.009 | 0.020 | 0.004 | 0.031 | 0.016 |
| *g__[Eubacterium]_brachy_group* | 0.000 | 0.000 | 0.017 | 0.021 | 0.018 | 0.034 | -0.033 | -0.005 | -0.017 |
| *g__unclassified_f__Streptosporangiaceae* | 0.017 | 0.010 | 0.000 | 0.000 | 0.001 | 0.010 | 0.011 | 0.025 | 0.017 |
| *g__norank_f__Bacteroidales_S24-7_group* | 0.017 | 0.024 | 0.000 | 0.000 | 0.019 | 0.034 | 0.001 | 0.036 | 0.017 |
| *g__Gallicola* | 0.016 | 0.020 | 0.000 | 0.000 | 0.019 | 0.034 | 0.004 | 0.032 | 0.016 |
| *g__Hyphomicrobium* | 0.014 | 0.014 | 0.001 | 0.002 | 0.024 | 0.044 | 0.004 | 0.024 | 0.013 |
| *g__Mycoplasma* | 0.014 | 0.011 | 0.001 | 0.003 | 0.005 | 0.014 | 0.004 | 0.022 | 0.013 |
| *g__Neochlamydia* | 0.014 | 0.008 | 0.001 | 0.003 | 0.005 | 0.014 | 0.005 | 0.020 | 0.013 |
| *g__Anoxybacillus* | 0.014 | 0.023 | 0.000 | 0.000 | 0.019 | 0.034 | 0.002 | 0.033 | 0.014 |
| *g__Solibacillus* | 0.014 | 0.022 | 0.000 | 0.000 | 0.006 | 0.014 | 0.003 | 0.032 | 0.014 |
| *g__Turicibacter* | 0.013 | 0.011 | 0.000 | 0.000 | 0.001 | 0.010 | 0.006 | 0.022 | 0.013 |
| *g__Jeotgalicoccus* | 0.012 | 0.015 | 0.000 | 0.000 | 0.006 | 0.014 | 0.003 | 0.025 | 0.012 |
| *g__Anaerosalibacter* | 0.012 | 0.010 | 0.000 | 0.000 | 0.001 | 0.010 | 0.005 | 0.019 | 0.012 |
| *g__Ammoniphilus* | 0.012 | 0.012 | 0.000 | 0.000 | 0.001 | 0.010 | 0.005 | 0.022 | 0.012 |
| *g__norank_f__Xanthomonadaceae* | 0.012 | 0.009 | 0.000 | 0.000 | 0.001 | 0.010 | 0.006 | 0.019 | 0.012 |
| *g__Devosia* | 0.011 | 0.008 | 0.000 | 0.001 | 0.010 | 0.023 | 0.005 | 0.016 | 0.011 |
| *g__Actinomadura* | 0.011 | 0.017 | 0.000 | 0.001 | 0.016 | 0.034 | 0.002 | 0.025 | 0.011 |
| *g__Atopostipes* | 0.012 | 0.017 | 0.000 | 0.000 | 0.006 | 0.014 | 0.002 | 0.026 | 0.012 |
| *g__unclassified_f__Intrasporangiaceae* | 0.011 | 0.011 | 0.000 | 0.000 | 0.001 | 0.010 | 0.004 | 0.020 | 0.011 |
| *g__norank_f__p35j06ok* | 0.011 | 0.019 | 0.000 | 0.000 | 0.006 | 0.014 | 0.001 | 0.027 | 0.011 |
| *g__Singulisphaera* | 0.011 | 0.012 | 0.000 | 0.000 | 0.006 | 0.014 | 0.003 | 0.020 | 0.011 |
| *g__norank_f__Mitochondria* | 0.011 | 0.015 | 0.000 | 0.000 | 0.019 | 0.034 | 0.002 | 0.024 | 0.011 |
| *g__unclassified_f__Paenibacillaceae* | 0.011 | 0.009 | 0.000 | 0.000 | 0.001 | 0.010 | 0.004 | 0.017 | 0.011 |
| *g__Flexivirga* | 0.010 | 0.006 | 0.000 | 0.000 | 0.006 | 0.014 | 0.005 | 0.015 | 0.010 |
| *g__norank_p__TM6__Dependentiae_* | 0.010 | 0.013 | 0.000 | 0.000 | 0.006 | 0.014 | 0.003 | 0.022 | 0.010 |
| *g__unclassified_f__Hyphomicrobiaceae* | 0.009 | 0.009 | 0.000 | 0.001 | 0.003 | 0.012 | 0.004 | 0.017 | 0.009 |
| *g__Bhargavaea* | 0.010 | 0.009 | 0.000 | 0.000 | 0.006 | 0.014 | 0.003 | 0.016 | 0.010 |
| *g__Tsukamurella* | 0.010 | 0.011 | 0.000 | 0.000 | 0.006 | 0.014 | 0.003 | 0.019 | 0.010 |
| *g__Thermoflavimicrobium* | 0.010 | 0.013 | 0.000 | 0.000 | 0.019 | 0.034 | 0.001 | 0.019 | 0.010 |
| *g__Pediococcus* | 0.010 | 0.011 | 0.000 | 0.000 | 0.006 | 0.014 | 0.002 | 0.018 | 0.010 |
| *g__Butyricicoccus* | 0.000 | 0.000 | 0.009 | 0.012 | 0.018 | 0.034 | -0.018 | -0.002 | -0.009 |
| *g__unclassified_o__Micrococcales* | 0.009 | 0.007 | 0.000 | 0.000 | 0.006 | 0.014 | 0.004 | 0.014 | 0.009 |
| *g__Tuberibacillus* | 0.009 | 0.005 | 0.000 | 0.000 | 0.001 | 0.010 | 0.005 | 0.013 | 0.009 |
| *g__Ktedonobacter* | 0.009 | 0.013 | 0.000 | 0.000 | 0.019 | 0.034 | 0.001 | 0.020 | 0.009 |
| *g__Symbiobacterium* | 0.008 | 0.008 | 0.000 | 0.000 | 0.006 | 0.014 | 0.003 | 0.015 | 0.008 |
| *g__unclassified_f__Acetobacteraceae* | 0.008 | 0.009 | 0.000 | 0.000 | 0.006 | 0.014 | 0.003 | 0.015 | 0.008 |
| *g__unclassified_o__Chlamydiales* | 0.008 | 0.007 | 0.000 | 0.000 | 0.001 | 0.010 | 0.004 | 0.013 | 0.008 |
| *g__Crossiella* | 0.008 | 0.010 | 0.000 | 0.000 | 0.019 | 0.034 | 0.002 | 0.016 | 0.008 |
| *g__norank_o__Solirubrobacterales* | 0.008 | 0.009 | 0.000 | 0.000 | 0.019 | 0.034 | 0.001 | 0.015 | 0.008 |
| *g__Virgibacillus* | 0.008 | 0.007 | 0.000 | 0.000 | 0.019 | 0.034 | 0.003 | 0.012 | 0.008 |
| *g__Dietzia* | 0.007 | 0.006 | 0.000 | 0.001 | 0.005 | 0.014 | 0.002 | 0.011 | 0.007 |
| *g__Smaragdicoccus* | 0.007 | 0.009 | 0.000 | 0.000 | 0.019 | 0.034 | 0.001 | 0.013 | 0.007 |
| *g__Thermobacillus* | 0.007 | 0.008 | 0.000 | 0.000 | 0.006 | 0.014 | 0.002 | 0.013 | 0.007 |
| *g__Agaricicola* | 0.007 | 0.005 | 0.000 | 0.000 | 0.006 | 0.014 | 0.003 | 0.011 | 0.007 |
| *g__Enteractinococcus* | 0.007 | 0.006 | 0.000 | 0.000 | 0.006 | 0.014 | 0.002 | 0.012 | 0.007 |
| *g__norank_f__288-2* | 0.006 | 0.005 | 0.000 | 0.000 | 0.006 | 0.014 | 0.003 | 0.010 | 0.006 |
| *g__Tepidimicrobium* | 0.006 | 0.004 | 0.000 | 0.000 | 0.006 | 0.014 | 0.004 | 0.009 | 0.006 |
| *g__Ruminiclostridium* | 0.000 | 0.000 | 0.006 | 0.009 | 0.018 | 0.034 | -0.014 | -0.002 | -0.006 |
| *g__Globicatella* | 0.006 | 0.006 | 0.000 | 0.000 | 0.019 | 0.034 | 0.002 | 0.011 | 0.006 |
| *g__Pseudoxanthomonas* | 0.006 | 0.007 | 0.000 | 0.000 | 0.006 | 0.014 | 0.002 | 0.011 | 0.006 |
| *g__norank_c__Bacilli* | 0.006 | 0.004 | 0.000 | 0.000 | 0.001 | 0.010 | 0.004 | 0.009 | 0.006 |
| *g__unclassified_f__Planococcaceae* | 0.006 | 0.010 | 0.000 | 0.000 | 0.019 | 0.034 | 0.001 | 0.014 | 0.006 |
| *g__Nitrolancea* | 0.006 | 0.006 | 0.000 | 0.000 | 0.006 | 0.014 | 0.002 | 0.011 | 0.006 |
| *g__Sphaerisporangium* | 0.006 | 0.004 | 0.000 | 0.000 | 0.006 | 0.014 | 0.003 | 0.009 | 0.006 |
| *g__Candidatus_Protochlamydia* | 0.006 | 0.007 | 0.000 | 0.000 | 0.006 | 0.014 | 0.002 | 0.011 | 0.006 |
| *g__norank_o__MBA03* | 0.006 | 0.006 | 0.000 | 0.000 | 0.001 | 0.010 | 0.002 | 0.011 | 0.006 |
| *g__Oceanobacillus* | 0.005 | 0.006 | 0.000 | 0.000 | 0.006 | 0.014 | 0.001 | 0.010 | 0.005 |
| *g__Streptosporangium* | 0.005 | 0.007 | 0.000 | 0.000 | 0.006 | 0.014 | 0.001 | 0.011 | 0.005 |
| *g__norank_f__0319-6M6* | 0.005 | 0.007 | 0.000 | 0.000 | 0.019 | 0.034 | 0.001 | 0.011 | 0.005 |
| *g__norank_o__Rhizobiales* | 0.005 | 0.003 | 0.000 | 0.000 | 0.006 | 0.014 | 0.003 | 0.007 | 0.005 |
| *g__Filobacterium* | 0.005 | 0.003 | 0.000 | 0.000 | 0.001 | 0.010 | 0.002 | 0.007 | 0.005 |
| *g__norank_o__C178B* | 0.004 | 0.004 | 0.000 | 0.000 | 0.006 | 0.014 | 0.002 | 0.007 | 0.004 |
| *g__Ruminiclostridium_1* | 0.004 | 0.004 | 0.000 | 0.000 | 0.019 | 0.034 | 0.002 | 0.007 | 0.004 |
| *g__Patulibacter* | 0.004 | 0.007 | 0.000 | 0.000 | 0.006 | 0.014 | 0.001 | 0.010 | 0.004 |
| *g__Ammoniibacillus* | 0.004 | 0.005 | 0.000 | 0.000 | 0.019 | 0.034 | 0.001 | 0.008 | 0.004 |
| *g__Salana* | 0.004 | 0.003 | 0.000 | 0.000 | 0.006 | 0.014 | 0.002 | 0.007 | 0.004 |
| *g__Actinoallomurus* | 0.004 | 0.005 | 0.000 | 0.000 | 0.006 | 0.014 | 0.001 | 0.008 | 0.004 |
| *g__Ornithinimicrobium* | 0.004 | 0.003 | 0.000 | 0.000 | 0.006 | 0.014 | 0.002 | 0.006 | 0.004 |
| *g__norank_f__Family_XVIII* | 0.003 | 0.003 | 0.000 | 0.000 | 0.019 | 0.034 | 0.001 | 0.006 | 0.003 |
| *g__norank_o__HTA4* | 0.003 | 0.004 | 0.000 | 0.000 | 0.006 | 0.014 | 0.001 | 0.007 | 0.003 |
| *g__norank_f__Planctomycetaceae* | 0.003 | 0.003 | 0.000 | 0.000 | 0.019 | 0.034 | 0.001 | 0.006 | 0.003 |
| *g__Friedmanniella* | 0.003 | 0.004 | 0.000 | 0.000 | 0.019 | 0.034 | 0.001 | 0.006 | 0.003 |
| *g__norank_c__JG30-KF-CM66* | 0.003 | 0.004 | 0.000 | 0.000 | 0.019 | 0.034 | 0.001 | 0.006 | 0.003 |
| *g__Anaerosporobacter* | 0.002 | 0.002 | 0.000 | 0.000 | 0.006 | 0.014 | 0.001 | 0.004 | 0.002 |

**Table S3** Differences between the HA and HB groups at the genus level (Wilcoxon rank-sum test, *P* < 0.05).

| Species name | HE-Mean (%) | HE-Sd (%) | RE-Mean (%) | RE-Sd (%) | *P* value | Corrected *p* value | Lower ci | Upper ci | Effectsize |
| --- | --- | --- | --- | --- | --- | --- | --- | --- | --- |
| *g__Deefgea* | 0.000 | 0.000 | 16.520 | 19.040 | 0.004 | 0.023 | -31.270 | -5.153 | -16.520 |
| *g__Parabacteroides* | 15.520 | 7.079 | 0.365 | 0.405 | 0.006 | 0.025 | 9.989 | 20.660 | 15.150 |
| *g__Lachnoclostridium* | 6.572 | 4.770 | 1.371 | 1.440 | 0.009 | 0.033 | 2.003 | 9.416 | 5.201 |
| *g__unclassified_f__Ruminococcaceae* | 6.551 | 2.179 | 1.180 | 0.981 | 0.006 | 0.025 | 3.534 | 7.116 | 5.371 |
| *g__Citrobacter* | 5.690 | 4.385 | 0.147 | 0.180 | 0.006 | 0.025 | 2.382 | 9.305 | 5.543 |
| *g__unclassified_f__Lachnospiraceae* | 4.825 | 2.913 | 0.709 | 1.189 | 0.015 | 0.049 | 1.653 | 6.710 | 4.116 |
| *g__Bacillus* | 5.393 | 2.136 | 0.007 | 0.014 | 0.005 | 0.024 | 3.842 | 7.108 | 5.387 |
| *g__Phascolarctobacterium* | 2.190 | 1.480 | 0.000 | 0.001 | 0.003 | 0.023 | 1.048 | 3.456 | 2.189 |
| *g__Odoribacter* | 0.000 | 0.000 | 1.712 | 2.148 | 0.004 | 0.023 | -3.502 | -0.541 | -1.712 |
| *g__Lactococcus* | 1.675 | 0.796 | 0.008 | 0.014 | 0.004 | 0.023 | 1.084 | 2.341 | 1.667 |
| *g__Alistipes* | 0.007 | 0.015 | 1.505 | 1.284 | 0.005 | 0.024 | -2.404 | -0.658 | -1.499 |
| *g__Gordonia* | 1.417 | 0.189 | 0.000 | 0.000 | 0.002 | 0.023 | 1.248 | 1.559 | 1.417 |
| *g__Fusobacterium* | 1.296 | 2.267 | 0.000 | 0.000 | 0.002 | 0.023 | 0.092 | 3.239 | 1.296 |
| *g__[Eubacterium]_coprostanoligenes_group* | 1.037 | 0.464 | 0.004 | 0.006 | 0.005 | 0.024 | 0.673 | 1.378 | 1.033 |
| *g__Ruminiclostridium_5* | 0.878 | 0.280 | 0.154 | 0.175 | 0.006 | 0.025 | 0.470 | 0.998 | 0.723 |
| *g__norank_o__Mollicutes_RF9* | 0.818 | 0.290 | 0.180 | 0.307 | 0.014 | 0.048 | 0.312 | 0.956 | 0.638 |
| *g__Escherichia-Shigella* | 0.984 | 0.841 | 0.001 | 0.002 | 0.004 | 0.023 | 0.409 | 1.804 | 0.982 |
| *g__Arcobacter* | 0.938 | 0.530 | 0.010 | 0.024 | 0.004 | 0.023 | 0.526 | 1.334 | 0.928 |
| *g__Rhodococcus* | 0.742 | 0.156 | 0.074 | 0.117 | 0.005 | 0.025 | 0.516 | 0.805 | 0.668 |
| *g__Peptoclostridium* | 0.785 | 1.611 | 0.000 | 0.000 | 0.002 | 0.023 | 0.036 | 2.230 | 0.786 |
| *g__Rikenella* | 0.000 | 0.000 | 0.724 | 1.062 | 0.004 | 0.023 | -1.494 | -0.129 | -0.724 |
| *g__Dysgonomonas* | 0.471 | 0.276 | 0.000 | 0.000 | 0.002 | 0.023 | 0.271 | 0.682 | 0.471 |
| *g__Coprobacillus* | 0.433 | 0.175 | 0.015 | 0.027 | 0.005 | 0.024 | 0.313 | 0.581 | 0.418 |
| *g__norank_f__Family_XIII* | 0.002 | 0.003 | 0.350 | 0.414 | 0.005 | 0.025 | -0.630 | -0.104 | -0.348 |
| *g__Chryseobacterium* | 0.000 | 0.000 | 0.351 | 0.705 | 0.012 | 0.041 | -0.885 | -0.021 | -0.351 |
| *g__Desulfovibrio* | 0.000 | 0.000 | 0.335 | 0.501 | 0.004 | 0.023 | -0.726 | -0.091 | -0.335 |
| *g__norank_f__FamilyI_o__SubsectionIV* | 0.284 | 0.449 | 0.000 | 0.000 | 0.002 | 0.023 | 0.063 | 0.680 | 0.284 |
| *g__norank_f__Clostridiales_vadinBB60_group* | 0.234 | 0.195 | 0.001 | 0.002 | 0.004 | 0.023 | 0.123 | 0.414 | 0.233 |
| *g__Microbacterium* | 0.212 | 0.097 | 0.011 | 0.028 | 0.004 | 0.023 | 0.139 | 0.293 | 0.200 |
| *g__Romboutsia* | 0.201 | 0.125 | 0.005 | 0.011 | 0.005 | 0.024 | 0.090 | 0.292 | 0.196 |
| *g__Akkermansia* | 0.000 | 0.000 | 0.186 | 0.257 | 0.012 | 0.041 | -0.396 | -0.032 | -0.186 |
| *g__Butyricimonas* | 0.000 | 0.000 | 0.169 | 0.254 | 0.004 | 0.023 | -0.364 | -0.039 | -0.169 |
| *g__Caproiciproducens* | 0.161 | 0.062 | 0.000 | 0.001 | 0.003 | 0.023 | 0.110 | 0.205 | 0.160 |
| *g__Weissella* | 0.159 | 0.181 | 0.001 | 0.002 | 0.004 | 0.023 | 0.028 | 0.323 | 0.158 |
| *g__Ruminococcaceae_UCG-013* | 0.159 | 0.053 | 0.000 | 0.000 | 0.002 | 0.023 | 0.118 | 0.200 | 0.159 |
| *g__norank_f__Coriobacteriaceae* | 0.133 | 0.058 | 0.022 | 0.027 | 0.006 | 0.025 | 0.065 | 0.161 | 0.111 |
| *g__Mycobacterium* | 0.121 | 0.046 | 0.018 | 0.036 | 0.009 | 0.032 | 0.060 | 0.147 | 0.104 |
| *g__Enterococcus* | 0.129 | 0.047 | 0.002 | 0.003 | 0.004 | 0.023 | 0.094 | 0.167 | 0.127 |
| *g__Streptococcus* | 0.125 | 0.078 | 0.001 | 0.001 | 0.004 | 0.023 | 0.069 | 0.192 | 0.124 |
| *g__Coprococcus_1* | 0.111 | 0.037 | 0.000 | 0.000 | 0.002 | 0.023 | 0.080 | 0.138 | 0.111 |
| *g__Candidatus_Soleaferrea* | 0.101 | 0.040 | 0.003 | 0.004 | 0.005 | 0.024 | 0.069 | 0.136 | 0.099 |
| *g__Eggerthella* | 0.082 | 0.062 | 0.000 | 0.000 | 0.002 | 0.023 | 0.038 | 0.135 | 0.082 |
| *g__unclassified_f__FamilyI_o__SubsectionIV* | 0.063 | 0.084 | 0.000 | 0.000 | 0.002 | 0.023 | 0.021 | 0.141 | 0.063 |
| *g__Stenotrophomonas* | 0.055 | 0.035 | 0.002 | 0.003 | 0.004 | 0.023 | 0.022 | 0.081 | 0.053 |
| *g__Methylobacterium* | 0.052 | 0.033 | 0.003 | 0.008 | 0.006 | 0.025 | 0.027 | 0.077 | 0.049 |
| *g__Oerskovia* | 0.054 | 0.024 | 0.000 | 0.001 | 0.003 | 0.023 | 0.035 | 0.072 | 0.053 |
| *g__Mucispirillum* | 0.000 | 0.000 | 0.054 | 0.059 | 0.004 | 0.023 | -0.096 | -0.018 | -0.054 |
| *g__unclassified_p__Firmicutes* | 0.054 | 0.047 | 0.000 | 0.000 | 0.002 | 0.023 | 0.029 | 0.097 | 0.054 |
| *g__Pseudochrobactrum* | 0.051 | 0.027 | 0.000 | 0.001 | 0.003 | 0.023 | 0.031 | 0.073 | 0.051 |
| *g__Ochrobactrum* | 0.050 | 0.005 | 0.000 | 0.000 | 0.002 | 0.023 | 0.046 | 0.054 | 0.050 |
| *g__Morganella* | 0.038 | 0.056 | 0.002 | 0.005 | 0.015 | 0.050 | 0.005 | 0.088 | 0.036 |
| *g__Rhizobium* | 0.032 | 0.019 | 0.004 | 0.008 | 0.008 | 0.032 | 0.010 | 0.043 | 0.028 |
| *g__Sphingobacterium* | 0.030 | 0.042 | 0.000 | 0.000 | 0.009 | 0.032 | 0.007 | 0.067 | 0.030 |
| *g__Empedobacter* | 0.030 | 0.050 | 0.000 | 0.000 | 0.009 | 0.032 | 0.003 | 0.075 | 0.030 |
| *g__Leuconostoc* | 0.028 | 0.021 | 0.000 | 0.001 | 0.003 | 0.023 | 0.016 | 0.047 | 0.028 |
| *g__unclassified_f__Micromonosporaceae* | 0.027 | 0.047 | 0.000 | 0.000 | 0.002 | 0.023 | 0.004 | 0.069 | 0.027 |
| *g__Cellulosimicrobium* | 0.027 | 0.021 | 0.000 | 0.000 | 0.009 | 0.032 | 0.010 | 0.043 | 0.027 |
| *g__Geobacillus* | 0.025 | 0.021 | 0.000 | 0.000 | 0.002 | 0.023 | 0.010 | 0.043 | 0.025 |
| *g__Anaerofustis* | 0.025 | 0.013 | 0.000 | 0.000 | 0.002 | 0.023 | 0.013 | 0.033 | 0.025 |
| *g__norank_f__Peptococcaceae* | 0.023 | 0.011 | 0.000 | 0.000 | 0.002 | 0.023 | 0.015 | 0.032 | 0.023 |
| *g__Catabacter* | 0.022 | 0.017 | 0.000 | 0.000 | 0.002 | 0.023 | 0.012 | 0.038 | 0.022 |
| *g__Planktothrix* | 0.021 | 0.018 | 0.000 | 0.000 | 0.002 | 0.023 | 0.008 | 0.036 | 0.021 |
| *g__Amaricoccus* | 0.018 | 0.014 | 0.001 | 0.002 | 0.003 | 0.023 | 0.009 | 0.029 | 0.017 |
| *g__unclassified_f__Enterobacteriaceae* | 0.017 | 0.011 | 0.001 | 0.002 | 0.004 | 0.023 | 0.007 | 0.025 | 0.017 |
| *g__Bosea* | 0.018 | 0.010 | 0.000 | 0.001 | 0.003 | 0.023 | 0.009 | 0.025 | 0.017 |
| *g__Nostoc* | 0.015 | 0.014 | 0.000 | 0.000 | 0.009 | 0.032 | 0.006 | 0.027 | 0.015 |
| *g__Kaistia* | 0.015 | 0.009 | 0.000 | 0.000 | 0.002 | 0.023 | 0.009 | 0.023 | 0.015 |
| *g__unclassified_f__Christensenellaceae* | 0.015 | 0.011 | 0.000 | 0.000 | 0.009 | 0.032 | 0.006 | 0.024 | 0.015 |
| *g__norank_f__Christensenellaceae* | 0.013 | 0.010 | 0.000 | 0.000 | 0.002 | 0.023 | 0.006 | 0.021 | 0.013 |
| *g__norank_o__HTA4* | 0.012 | 0.005 | 0.000 | 0.000 | 0.002 | 0.023 | 0.008 | 0.015 | 0.012 |
| *g__Paenibacillus* | 0.010 | 0.004 | 0.001 | 0.002 | 0.004 | 0.023 | 0.005 | 0.012 | 0.008 |
| *g__Lactobacillus* | 0.008 | 0.008 | 0.000 | 0.000 | 0.002 | 0.023 | 0.004 | 0.015 | 0.008 |
| *g__norank_p__Saccharibacteria* | 0.008 | 0.008 | 0.000 | 0.000 | 0.009 | 0.032 | 0.002 | 0.014 | 0.008 |
| *g__Dietzia* | 0.005 | 0.004 | 0.000 | 0.001 | 0.003 | 0.023 | 0.002 | 0.009 | 0.005 |
| *g__Ruminococcaceae_UCG-005* | 0.006 | 0.005 | 0.000 | 0.000 | 0.009 | 0.032 | 0.002 | 0.009 | 0.006 |
| *g__Patulibacter* | 0.004 | 0.003 | 0.000 | 0.000 | 0.009 | 0.032 | 0.002 | 0.006 | 0.004 |
| *g__Candidatus_Protochlamydia* | 0.004 | 0.003 | 0.000 | 0.000 | 0.009 | 0.032 | 0.002 | 0.006 | 0.004 |
| *g__unclassified_o__Lactobacillales* | 0.003 | 0.002 | 0.000 | 0.000 | 0.009 | 0.032 | 0.001 | 0.004 | 0.003 |
